# Single-walled and multi-walled carbon nanotubes induce sequence-specific epigenetic alterations in 16 HBE cells

**DOI:** 10.18632/oncotarget.24866

**Published:** 2018-04-17

**Authors:** Manosij Ghosh, Deniz Öner, Radu C. Duca, Bram Bekaert, Jeroen A.J. Vanoirbeek, Lode Godderis, Peter H.M. Hoet

**Affiliations:** ^1^ KU Leuven, Department of Public Health and Primary Care, Centre Environment and Health, B-3000 Leuven, Belgium; ^2^ Idewe, External Service for Prevention and Protection at Work, B-3001 Heverlee, Belgium; ^3^ Forensic Biomedical Sciences, Department of Imaging and Pathology, KU Leuven, University of Leuven, Leuven, Belgium; ^4^ Department of Forensic Medicine, Laboratory of Forensic Genetics and Molecular Archaeology, University Hospitals Leuven, Leuven, Belgium

**Keywords:** nanotoxicology, MWCNT, SWCNT, epigenetics, DNA methylation

## Abstract

Recent studies have identified carbon nanotube (CNT)-induced epigenetic changes as one of the key players in patho-physiological response. In the present study, we investigated whether CNT exposure is associated with epigenetic changes in human bronchial epithelial cells (16 HBE), *in vitro*. We focused on global DNA methylation, methylation of *LINE-1* elements and promoter sequence of twelve functionally important genes (*SKI, DNMT1, HDAC4*, *NPAT, ATM, BCL2L11, MAP3K10, PIK3R2, MYO1C, TCF3, FGFR 1* and *AGRN*). Additionally, we studied the influence of CNT exposure on miRNA expression. Using a LC-MS/MS method and pyrosequencing for *LINE-1*, we observed no significant changes in global DNA methylation (%) between the concentrations of multi-walled and single-walled CNT (MWCNT and SWCNT, respectively). Significant changes in sequence-specific methylation was observed in at least one CpG site for *DNMT1* (SWCNT), *HDAC4* (MWCNT), *NPAT/ATM* (MWCNT and SWCNT), *MAP3K10* (MWCNT), *PIK3R2* (MWCNT and SWCNT) and *MYO1C* (SWCNT). While changes in DNA methylation of the genes were relatively small, these changes were associated with changes in RNA expression, especially for MWCNT. However, the study did not reveal any significant alteration in the miRNA expression, associated with MWCNT and SWCNT exposure. Based on our results, mainly MWCNT influence DNA methylation and expression of the studied genes and could have significant impact on several critical cellular processes.

## INTRODUCTION

Over the past decade, several aspects of carbon nanotube (CNT)-induced toxicity have been described in both *in vitro* and *in vivo* studies [1–7]. Many of these studies have provided conclusive evidences on cytotoxicity, inflammatory response, and genotoxicity. Moreover, in some CNTs, molecular signatures, similar to those observed in cancer, have been identified. Both multi-walled carbon nanotubes (MWCNT) and single-walled carbon nanotubes (SWCNT) have been shown to induce cell cycle arrest and activation of NF-κB, AP-1, AKT and MAPK signalling pathways [8–11]. Polimeni et al. [12]. reported that MWCNT could promote TGF-β secretion and AKT activation, along with the incidence of pulmonary fibrosis in C57BL/6 mice. Vietti et al. [13]. elaborately reviewed the data on alterations in key pathways, associated with CNT-induced pro-inflammatory and pro-fibrotic outcomes.

Additionally, a limited number of studies have reported adverse effects of CNT exposure in human populations. Of these studies, some have reported significant alterations in immunological markers, miRNA, and mRNA expression. Significant changes in non-coding RNA and mRNA, with key roles in cell cycle regulation-progression, apoptosis and cell proliferation have been reported for workers exposed to MWCNT [14]. Increased levels of KL-6/TGF-β have been reported in MWCNT-exposed workers [15]. Vlaanderen et al. [16] also reported adverse effect on immunological markers in workers exposed to MWCNT. In the same population, we observed significant changes in DNA methylation in a number of CpG sites, located in the promoter regions of *DNMT1*, *HDAC4*, *SKI*, *ATM/NPAT* genes [17].

Based on some of these data (*in vitro*, *in vivo* and in humans), most CNT have been classified as Group 3 (“Not classifiable as to its carcinogenicity to humans”; MWCNT and SWCNT) and MWCNT-7 has been classified as Group 2B (“Possibly carcinogenic to humans”) by the IARC (International Agency for Research on Cancer). However, these studies provide only partial mechanistic explanation as to how the CNT could result in possible carcinogenic response. Changes in epigenetic signature and noncoding RNA, in cancer initiation and progression are well established. Of the epigenetic alterations, methylation of DNA is crucial to cellular processes including chromatin folding and gene expression. Aberrant methylation signatures associated with altered gene expression and dysregulation of key pathways have been observed in cancer [18–21]. In this context, some recent studies [22–26] including that by our group [17, 27–29] have identified epigenetic changes as one of the potential players in CNT-induced patho-physiological response. In our previous study in human monocytes (THP-1) [28] and bronchial epithelial cells (16-HBE) [29], we observed differential methylation of genes in PI3K-AKT-mTOR pathway, JAK-STAT pathway, MAPK pathway, cell cycle, VEGF pathways. The epigenetic changes in pathways from our study were in agreement with that reported by other studies using other endpoints [8, 10, 11].

Since CNTs are likely to deposit on the lung tissue, studying DNA methylation and subsequent gene expression alterations in these cells will elucidate possible forthcoming adverse effects upon exposure.

Hence, in the present study we have directed our efforts to identification of DNA methylation changes, as possible signatures of CNT exposure and response. We have studied changes in global DNA methylation/demethylation processes (using LC-MS/MS), and methylation of long interspersed nuclear element (*LINE-1*). Additionally, to identify specific DNA methylation markers, we studied sequence-specific changes in promoter region of key genes (*SKI*, *DNMT*1, *HDAC*4, *NPAT*, *ATM*, *BCL2L11*, *MAP3K10*, *PIK3R2*, *MYO1C*, *TCF3*, *FGFR*1, *AGRN*) associated with DNA damage repair response and apoptosis (*ATM*, *NPAT*, *BCL2L11*, *MAP3K10*), epigenetic regulation (*DNMT1*, *HDAC4*), among others. To use such markers efficiently, we studied expression of these genes. Among the non-coding RNAs, miRNAs have often been associated with gene regulation and disease phenotypes and are often considered optimal markers. Hence, we studied the changes in CNT- induced miRNA expression profile. We believe that the result obtained from the study will potentially improve the understanding of CNT-induced toxic response.

## RESULTS

### Characterization of particles

**Figure 1:**
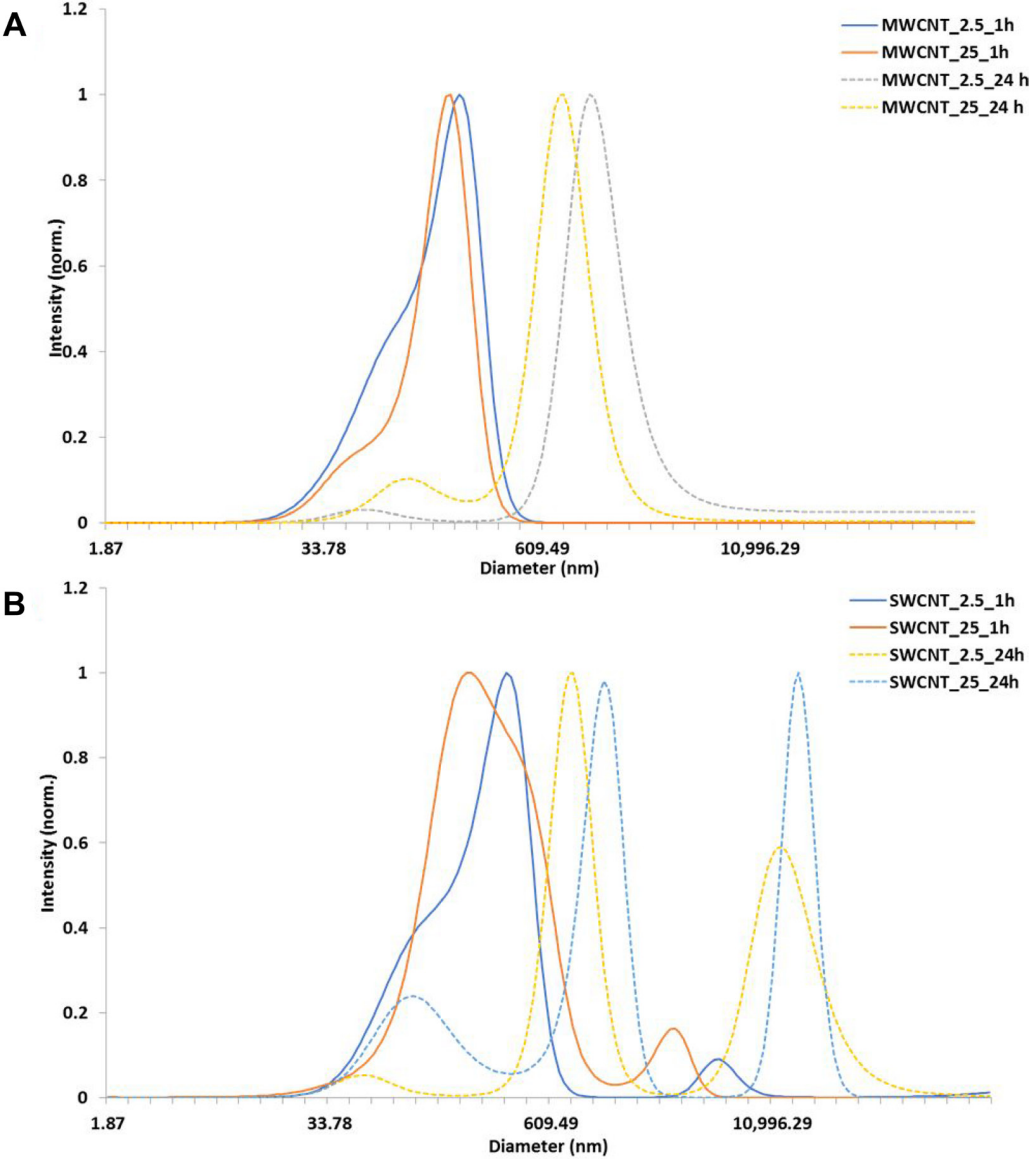
Characterization of carbon nanotubes in exposure medium (**A**) DLS measurements of MWCNT (NM 400), (**B**) DLS of SWCNT in cell culture media at 1 h and 24 h.

The CNT used in the present study were well-characterized reference materials from Joint Research Centre (JRC, European Commission) and National Institute of Standards and Technology (NIST, USA). Characterizations of the particle have been published elsewhere [28]. For the present study, we characterized the CNTs for primary shape using Transmission electron microscopy (TEM). Dynamic light scattering (DLS) measurements were performed for the exposure concentrations at two different time points, results of which have been represented in Figure 1A, 1B. While DLS is not an ideal method to determine hydrodynamic diameter for fibres such as CNT, this data has only been used as representative of aggregation state over time. The DLS measurements indicated significant increase in agglomeration at 24 h compared to that at 1 h.

### Global DNA methylation

**Figure 2:**
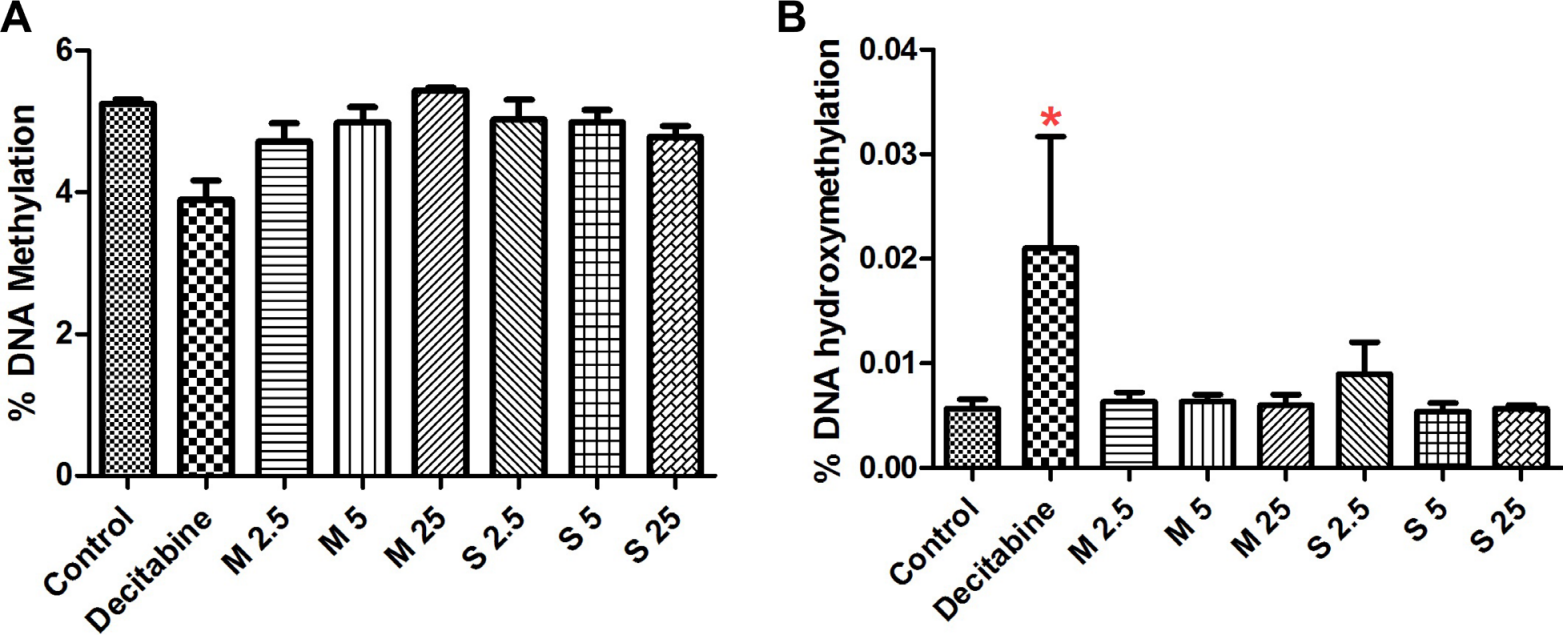
Effect of CNT exposure on global (**A**) DNA methylation and (**B**) hydroxy-methylation; numbers followed by M and S represents concentrations (µg/ml) of MWCNT and SWCNT respectively; bars represent mean ± SEM; ^*^P < 0.05.

The results of % DNA methylation [5-methylcytosine residues (5-mC)] and % DNA hydroxymethylation [5-hydroxymethylcytosine residues (5-hmC)] are presented in Figure 2. We did not observe any significant changes in mean value of global DNA methylation (%) and hydroxymethylation (%) for MWCNT (% 5-mC- 4.71- 5.433; % 5-OHmC- 0.0060- 0.0064) and SWCNT (% 5-mC- 4.78- 5.03; % 5-OHmC- 0.0049-0.009) treatments, compared to control samples (%5-mC -5.24; % 5-OHmC- 0.006).

### Methylation of repetitive element LINE-1

**Figure 3:**
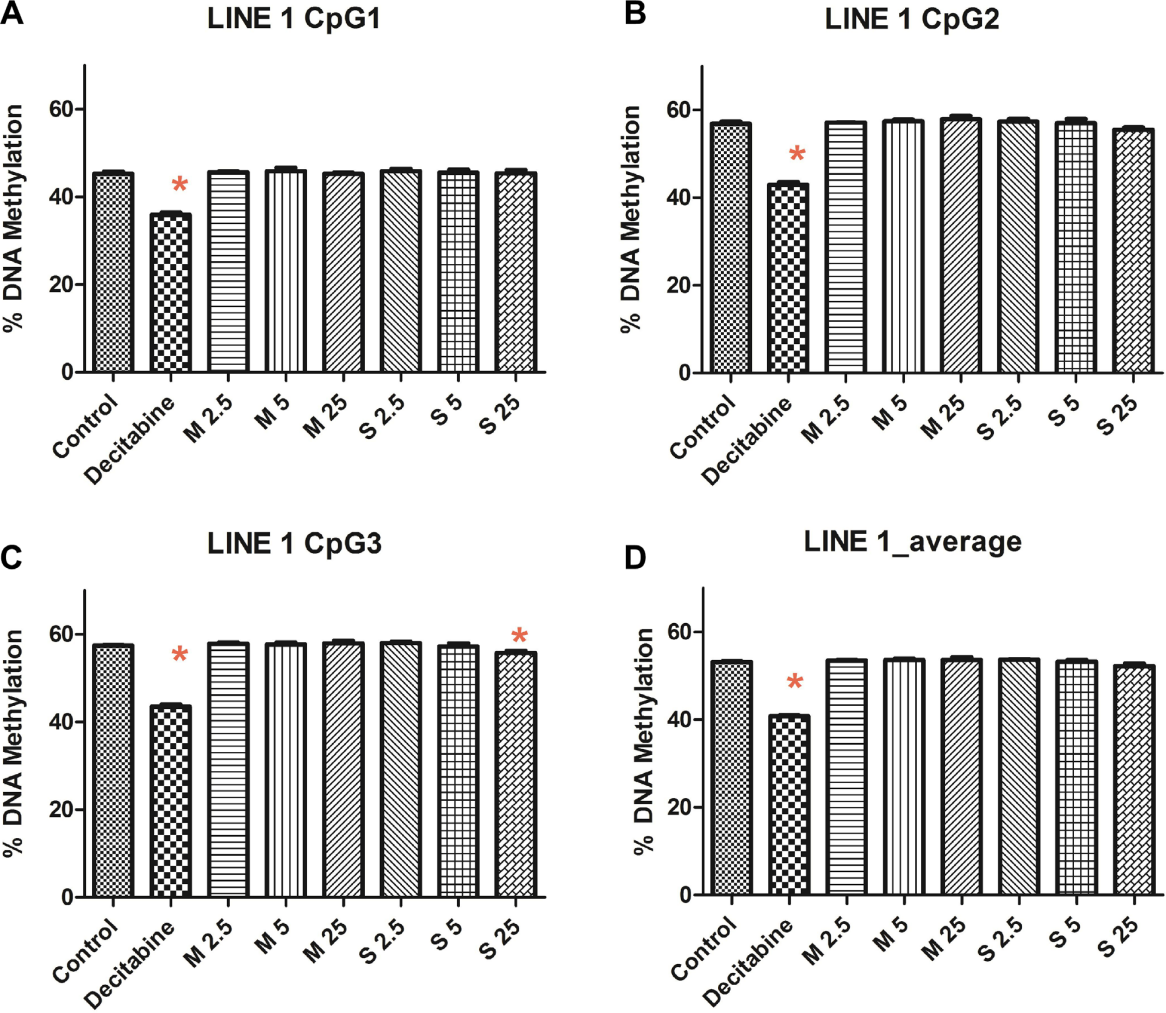
*LINE-1* methylation at (**A**) CpG position 1, (**B**) CpG position 2, (**C**) CpG position 3 and (**D**) average methylation of CpG sites; numbers followed by M and S represents concentrations (µg/ml) of MWCNT and SWCNT respectively; bars represent mean ± SEM; ^*^P < 0.05.

We quantified the *LINE-1* methylation levels using pyrosequencing. The percentage of *LINE-1* methylation remained mostly unaltered (Figure 3) for MWCNT (53.63 ± 0.5) and SWCNT (53.21 ± 0.77), compared to control (53.15 ± 0.17), while significant changes were observed for decitabine (positive control, 40.74 ± 0.54).

### Sequence specific methylation and changes in RNA expression

Expression of several genes selected for the study were significantly altered for MWCNT exposure (*BCL2L11*, *MYO1C*, *AGRN*, *SKI*, *ATM*, *NPAT*, *PIK3R2*, *TCF3*, *and FGFR1)* compared to the vehicle control (Table 1).

**Table 1:** Summary of RNA expression data for the genes studied for pyrosequencing assays

	Control Vs Decitabine		Control Vs MWCNT		Control Vs SWCNT	
	***logFC***	***FDR***	***logFC***	***FDR***	***logFC***	***FDR***
**AGRN**	0.592	0.369	**0.557**	**> 0.001**	0.057	0.821
**ATM**	0.068	0.906	**-0.430**	**> 0.001**	–0.039	0.886
**BCL2L11**	0.135	0.921	–**0.357**	**> 0.001**	–0.244	0.091
**DNMT1**	–0.170	0.453	0.127	0.147	0.045	0.811
**FGFR1**	–0.009	0.989	**0.315**	**0.001**	0.103	0.615
**HDAC4**	–0.469	0.509	0.026	0.871	0.006	0.986
**MAP3K10**	0.015	0.989	0.180	0.121	–0.184	0.385
**MYO1C**	0.367	0.781	**0.206**	**0.015**	0.032	0.874
**NPAT**	–0.044	0.972	–**0.231**	**0.004**	0.033	0.859
**PIK3R2**	–0.020	0.979	**0.275**	**0.001**	–0.038	0.843
**SKI**	0.806	0.126	**0.243**	**0.005**	0.027	0.902
**TCF3**	0.062	0.942	**0.346**	**> 0.001**	0.054	0.777

Values in bold represent statistically significant values, log Fold Change (logFC), false discovery rate (FDR).

**Figure 4:**
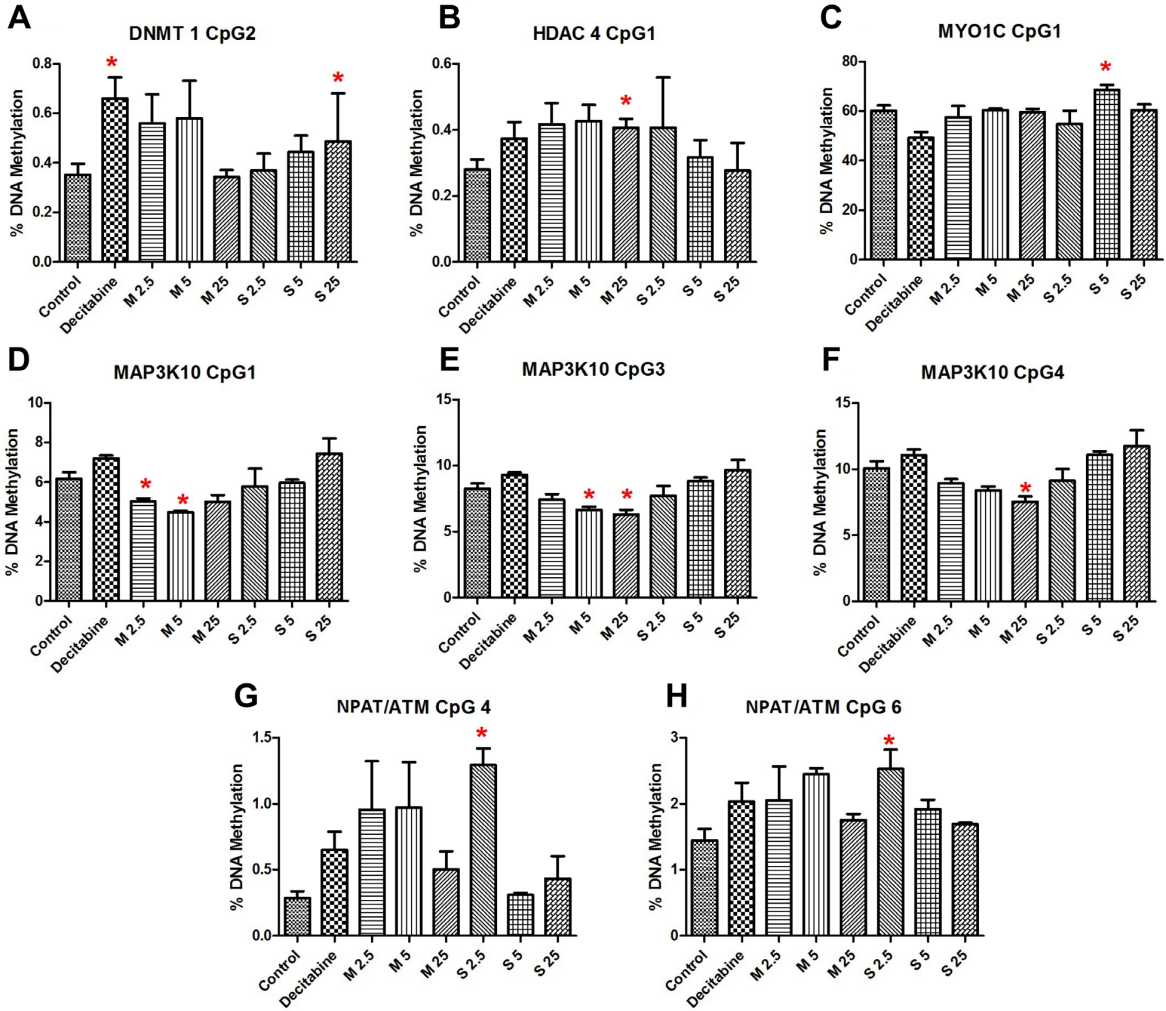
Results showing significant changes in sequence specific methylation of (**A**) *DNMT1* CpG 2, (**B**) *HDAC4* CpG 1, (**C**) *MYO1C* CpG 1, (**D**) *MAP3K10* CpG 1, (**E**) *MAP3K10* CpG 3, (**F**) *MAP3K10* CpG 4, (**G**) *NPAT/ATM* CpG 4, (**H**) *NPAT/ATM* CpG 6; bars represent mean ± SEM; ^*^P < 0.05. Numbers followed by M and S represents concentrations (µg/ml) of MWCNT and SWCNT respectively.

The results of pyrosequencing analysis have been presented in Supplementary Table 2. Significant changes in methylation were observed (Figure 4) for the genes either for MWCNT exposure (*HDAC4*, *MAP3K10*), SWCNT exposure (*MYO1C*, *NPAT*/*ATM*, *DNMT1*), or average methylation for both (*PIK3R2*). While some of the CpG sites were hypomethylated others were hypermethylated, often within the same gene promoter region. For instance, CpG4 and CpG6 of *ATM/NPAT* promoter were hypermethylated. Interestingly, these CpG sites were also hypermethylated for decitabine (a known hypomethylating agent). No clear dose response pattern could be observed.

### Effect on miRNA expression

**Figure 5:**
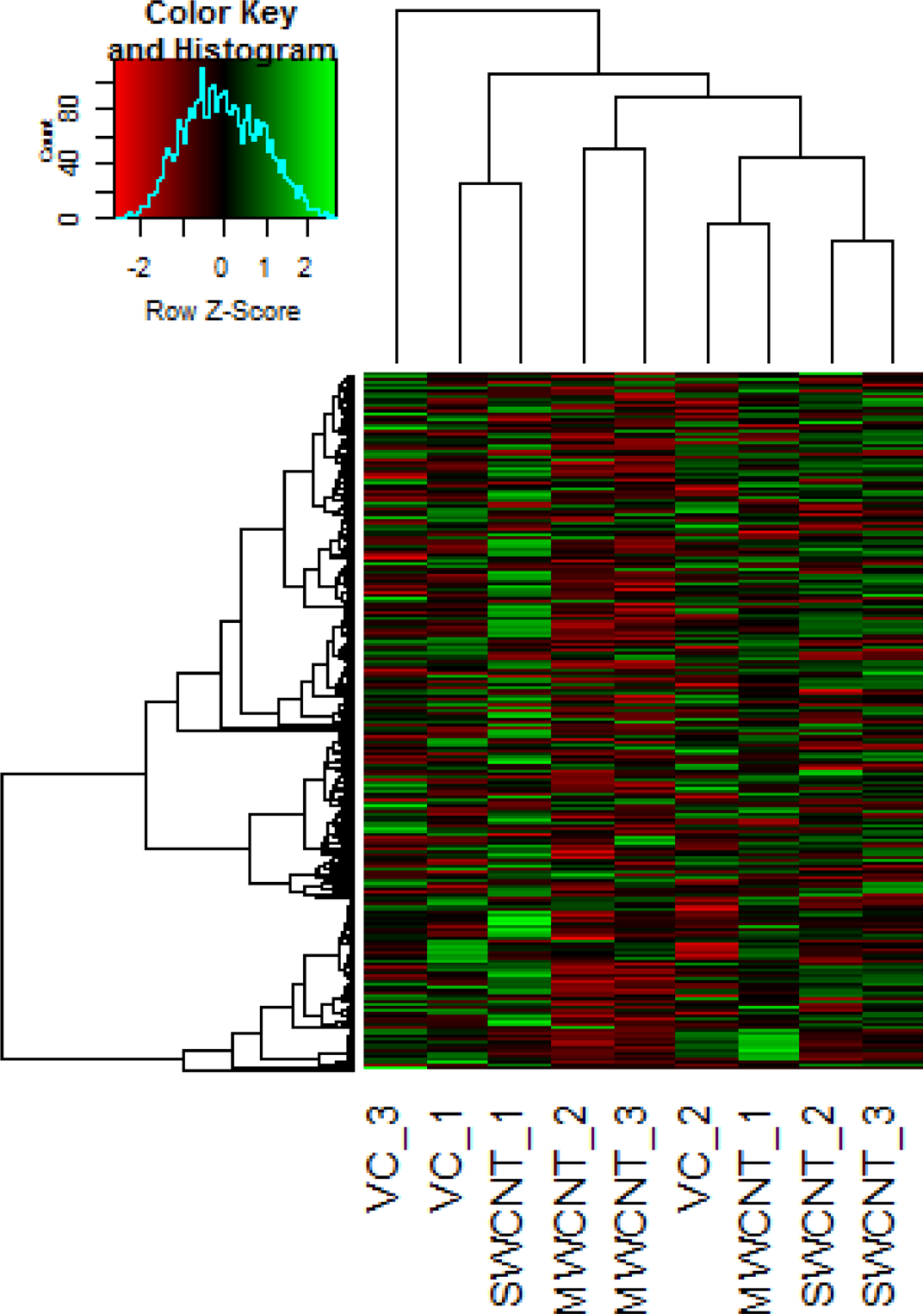
Representative heat map obtained by unsupervised hierarchical clustering of the miRNA expression data; each row represents a miRNA and each column represents a sample VC_1, VC_2 and VC_3 represent the vehicle control samples, MWCNT_1, MWCNT_2, MWCNT_3 represents multi walled carbon nanotube exposed samples and SWCNT_1, SWCNT_2, SWCNT_3 represents single walled carbon nanotube exposed samples. miRNA clustering tree is shown on the left, and the sample clustering tree appears at the top. The colour scale shown at the top illustrates the relative expression level of a miRNA in the certain slide: green, high relative expression level; red, low relative expression level.

Considering a fold change of > 2, no significant changes (FDR corrected *p*-value < 0.05) in expression of miRNA (Figure 5) were observed in the cells exposed to MWCNT and SWCNT (Supplementary Figure 3; Supplementary Table 3). Unsupervised hierarchical clustering of samples using the expression of the miRNA (*n =* 323), indicated one main cluster comprising the control samples as well as cells exposed to SWCNT and MWCNT.

## DISCUSSION

Several studies have clearly indicated the ability of CNT to induce DNA damage. Studies that are more recent have reported the effect of CNT on DNA damage repair pathways, post-transcriptional modifications, and dysregulation of genes involved in several critical pathways. Few recent studies have attributed CNT induced epigenetic alterations behind the dysregulation. Most of these signatures bear considerable resemblance to hallmarks of cancer related pathways. Some of the CNTs in use/production have shown evidence of potential carcinogenic response in animal studies. In a published study from our group, we observed, using femtosecond pulsed laser microscopy, localization of SWCNT and MWCNT at nuclear level after 24 h exposure. Based on the premise of potential carcinogenicity and evidence of nuclear localization, we designed a study to evaluate changes in global DNA methylation/hydroxymethylation levels in 16 HBE cells, *in vitro*. Additionally, we investigated sequence-specific methylation changes in *LINE-1* elements and promoter specific methylation and expression of 12 critical genes involved in DNA damage repair and related pathways.

Altered global DNA methylation has been identified in several diseases [30, 31]. Here, we studied the changes in methylation (5-mC) induced by MWCNT and SWCNT; however, we did not observe significant changes. Additionally, due to the newly identified role of demethylation pathways in cancer [32, 33], we studied hydroxymethylation (5-hmC) in the CNT exposed cells. No significant changes in the 5-mC levels were observed either. We subsequently studied the methylation levels of *LINE-1*, which is considered a good indicator of global DNA methylation [34]. Sequence specific analysis of *LINE-1* did not reveal significant changes in methylation, except for a single concentration of SWCNT (25 µg/ml) at a CpG position 3. It must be mentioned that aberrant methylation of *LINE-1* has been observed in several diseases [35–37]. From our previous study [28] in monocytic cells and bronchial epithelial cell, despite absence of global methylation changes, we observed methylation changes in gene promoter region of several genes. Hence, in addition to global methylation changes, we wanted to see if certain genes with important role in cellular processes were differentially methylated.

Of the genes selected, we studied the sequence-specific methylation changes in bidirectional promoter for *ATM* and *NPAT* genes. *ATM* is important for an efficient DNA damage response (DDR) mechanism, which involves recruitment of early responders like phosphatidylinositol 3-kinase-related kinases (PIKK) - ataxia-telangiectasia mutated (*ATM*). *ATM* codes for serine-threonine kinase which acts by phosphorylating several important genes, ultimately leading to cell cycle arrest, DNA repair, and/or apoptosis. We observed significant hypermethylation of the region studied, significant for some concentrations of SWCNT. RNA expression was significantly reduced by MWCNT for *ATM* (*P <* 0.001 FDR corrected) and *NPAT* (*P <* 0.001, FDR corrected) genes. However, the changes in methylation of the sequence in question were not correlated with that of the RNA expression. CpG hypermethylation in *ATM* gene was of key interest for the present study as it has also been observed for CNT exposure in mice lungs by our group [27]. Interestingly, significant hypermethylation of CpG position 6, also seen in the present study, was observed for the same sequence region in our study on MWCNT-exposed workers [17]. Additionally, literature clearly reveals that *ATM* expression is downregulated in several forms of cancer including that of breast and lung [38–40]. In the context of epigenetic regulation however, some studies have shown promoter hypermethylation to be associated with reduced ATM expression [41, 42], while other studies have shown no evidence of epigenetic silencing of *ATM* gene [43, 44]. Based on the results we propose that CNT-induced downregulation of ATM expression and aberrant promoter methylation might be responsible for reduced DDR efficiency.

With significant functional and sequence similarities to the PIKKs, phosphatidylinositol - 3 kinases (PI3Ks) play key role in signal transduction, essential for cellular functions including cell growth, survival and apoptosis. PI3Ks in combination with protein kinase B (*AKT*) and *mTOR* (mammalian target of rapamycin) also known as the *PI3K*/*AKT* signalling pathway is crucial to many aspects of cell growth/survival and apoptosis, and subsequently cancer [45–47]. PI3K are heterodimers consisting of a p85 regulatory subunit and a p110 catalytic subunit. P85 is encoded by *PI3KR1* (p85α) and *PI3KR2* (p85β) forming the heterodimers. Mutations of *PIK3R1* and/or *PIK3R2* are also found in cancer cells resulting in the altered expression of PI3K [48, 49]. Therefore, we studied the epigenetic regulation of *PIK3R2/p85*β expression, which was significantly upregulated for MWCNT-treated cells (FDR corrected *P <* 0.001) though no significant difference in DNA methylation values between exposed and control samples were observed. Higher expression *PIK3R2* has been observed in lung squamous cell carcinoma as compared to normal tissue [50]. Since, increase in *PIK3R2* expression has been reported in several cancers [48, 51] we believe the findings of MWCNT are of considerable significance.

Myosin-1C (*MYO1C*), another important gene in the *PI3K*/*AKT* signalling pathway, is known to bind with PIP2 and potentially acts as a tumour suppressor [52, 53]. *MYO1C* has also a role in glucose uptake and cell cycle progression. In our study, the expression of *MYO1C* was significantly altered for MWCNT (FDR corrected *P* = 0.015), yet no association with promoter methylation was observed. Interestingly, in the study by Oldfors et al., [52] no evident association between reduced MYO1C expression and promoter methylation were observed in endometrial carcinoma.

*MAP3K10* showed significant change in DNA methylation patterns for MWCNT exposure. *MAP3K10* (*MLK2* or *MST*) is serine/threonine kinase which plays critical role in the JNK signalling cascade, by a series of phosphorylation and is significant in cell proliferation and apoptosis [54]. Significant hypomethylation of CpGs 3, 4 and 5 in the selected promoter sequence was observed for MWCNT (25 µg/ml); however, no significant changes were observed for SWCNT. The changes in methylation were not reflected in the RNA expression data, as only a non-significant increase in expression was observed for MWCNT (1.6-fold, FDR corrected *P* = 0.121).

Recruitment of acetyltransferases and deacetylase enzymes are a key to an efficient DDR mechanism as well as other critical cellular processes. *HDACs* associated with the DDR mechanism are mostly recruited post repair, where it plays key role in chromatin restoration. The role of HDACs in cancer has been clearly established by several studies [55, 56]. *HDAC4*, a class IIa *HDAC*, is one of the critical components of the DDR pathway that acts together with p53-binding protein 1 (*53BP1*) [57, 58]. We studied RNA expression of *HDAC4*, but no significant alterations were induced by MWCNT or SWCNT. However, err hypermethylation of CpG1 of the studied sequence was observed, significant for MWCNT treatment concentration 25 µg/ml. While the impact of methylation was not observed on the expression, possible adverse outcome cannot be ruled out based on spatial and temporal influence of DNA methylation on gene regulation and expression.

Cellular response to DNA damage also involves series of well synchronized events involving DNA methyltransferases (DNMTs). While *DNMTs* are key to establishing and maintaining DNA methylation patterns, contributing to chromatin structure and genome stability, evidences have suggested that some of the *DNMTs,* specifically *DNMT1* could be directly involved in DDR in methylation independent manner [59, 60]. Evidence suggests dysregulation of DNMT expression in cancers [61, 62]. In this study, we investigated promoter methylation and expression of DNMT1 to understand the impact of CNT exposure. We did not observe a significant change in RNA expression, on the contrary a significant hypermethylation of one CpG site in the promoter sequence was observed for SWCNT treatment.

Transcription factor 3 (TCF3/E2A) is involved in regulation of p21 and PUMA (p53 upregulated modulator of apoptosis), and plays significant role in determining p53 mediated apoptotic response. TCF3 was of additional interest as it was differentially methylated in monocytic cells, as reported in our previous study [28]. While an overall hypomethylation of promoter region was observed for MWCNT (0.53 ± 0.10) and SWCNT (0.42 ± 0.13) compared to the negative control (0.79 ± 0.52), the change was not significant in the present study. The expression of *TCF3* however, was significantly altered in MWCNT-treated cells (*P <* 0.001 FDR corrected), while it remained mostly unaltered for SWCNT treatment (*P =* 0.77, FDR corrected). Upregulation of TCF3 expression, associated with promoter hypomethylation is in accordance with previously published literature observed in colorectal cancer [63].

DNA damage may lead to apoptosis either by extrinsic (death receptor) or intrinsic (mitochondria) pathways. While the role of MAPK and TGF-beta signalling pathways are evident for CNT [64], there is growing evidence of CNT-induced mitochondrial disruption and apoptosis. The intrinsic apoptotic pathway is usually activated by DNA damage and can be activated by caspase 8. One of the central genes in the BCL2 family is *BCL2L11/BIM*, which encodes *BCL2L11*, a ‘BH3-only protein’ member of the BCL2 family. In our study, we observed a significant down regulation of *BCL2L11* (*P <* 0.001 FDR corrected) for MWCNTexposure. Hypermethylation of the promoter sequence was observed for all the treatment concentrations of MWCNT and SWCNT, admittedly it was not significant. Lower expression of *BCL2L11* associated with DNA hypermethylation has been observed in chronic myeloid leukaemia [65]. In another study, hypermethylation of *BCL2L11* was observed after exposure to bisphenol A [66]. Fish [67] also observed promoter hyper-methylation *BCL2L11* in lung tumours.

Amplification and dysregulation of FGR family and specifically *FGFR1* has been observed in small cell carcinoma and other forms of cancer [68–70]. In our study, we observed a significant (FDR corrected *P <* 0.001) upregulation of *FGFR1* expression for MWCNT treatment, which was not associated with DNA methylation of the promoter sequence (*R*2 = 0.116). While a hypomethylation of CpG positions 1 and 2 were observed for MWCNT, the change was not significant. *FGFR1* gene was also found to be significantly hypo- methylated by CNT in our previous study on monocyte cell- THP-1 [28]. In another study, Goldstein et al. [69]. reported overexpression of *FGFR1* associated with hypomethylation of a CpG island upstream of exon 1. We also studied the changes in methylation and expression of SKI gene, which plays a critical role in TGF-beta signalling repression [71]. Changes in signalling pathway (TGF-β, Akt/GSK-3β, SNAIL-1) has also been reported for BEAS-2B cells [12]. In our study however, methylation of the CpG sites remained unaffected.

Taken together, in our study, we did not observe any significant changes in level of global DNA methylation. While some sequence specific methylation changes were observed, the changes were relatively small. Some of these changes were found to be associated with changes in RNA expression. Based on literature it is now evident that CNTs may induced epigenetic changes, however the results are not always uniform. *In vitro* exposure to carbon-based nanoparticles (carbon black, short MWCNTs and SWCNTs) induced global hypermethylation in A549 cells [26], while our previously published studies indicated no changes at global levels [28, 29]. Differences in global DNA methylation were found to be cell type specific by Chatterjee et al., [23], where MWCNT induced global DNA hypermethylation in HepG2 cells, whereas it induced hypomethylation in BEAS-2B cells. Epigenome wide analysis of methylation in THP-1 cells [28], revealed gene promoter-specific hypomethylation of more than 1000 genes. Our study in 16-HBE cell, also revealed that a large number of genes were hypomethylated at promoter sites after exposure to MWCNT and SWCNT [29]. In the study [29] we observed the changes in methylation to be associated with changes in expression of *ATM, SKI, GSTP1, NF1* among others. Epigenome wide study by Sierra et al. [25], also indicated hypomethylation of 755 CpG sites in BEAS-2B cells exposed to MWCNTs. Sierra et al., [25], also reported that these changes were concentration and time dependent, with CpG sites more differentially hypomethylated at 4 weeks compared to 2 weeks exposure. Relatively fewer studies have investigated the effect of CNT exposure in rodent model. Intra-tracheal administration of CNTs have been shown to induce epigenetic changes in mice lung tissue, where changes in gene promoter methylation was observed for ATM gene [27]. In addition to global DNA hypomethylation, the study by Brown et al., [22] in C57BL/6 mice also reported promoter specific methylation of IFN-γ and TNF-αgenes in the lung tissue of MWCNT exposed mice. It may be important to note, the results of our present study are consistent with our study in human population [17], where sequence specific DNA methylation changes (*DNMT1*, *ATM*, *SKI*, *HDAC4*) were observed in MWCNT exposed workers, despite no changes in global DNA methylation.

**Figure 6:**
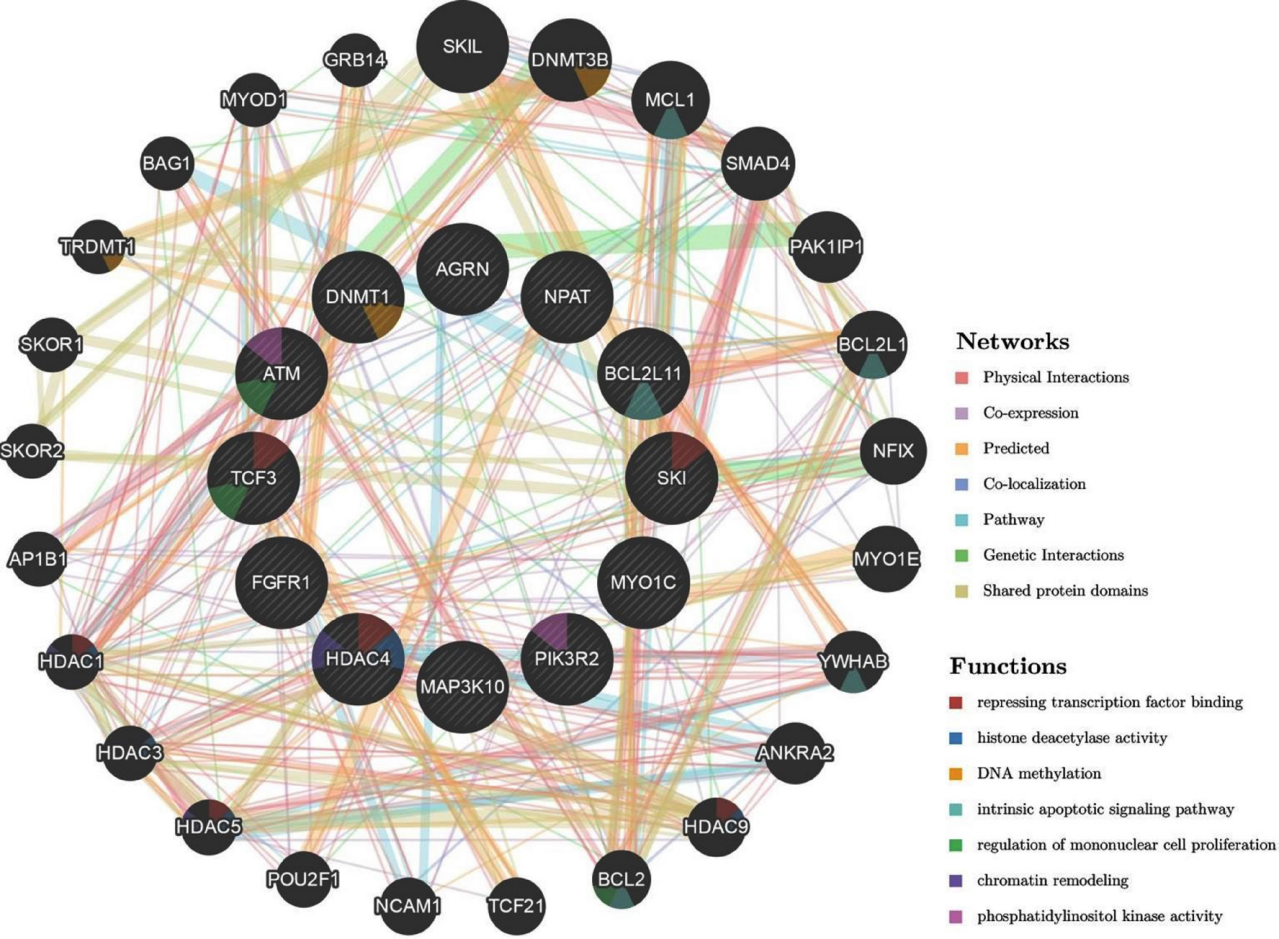
Schematic representation of the interaction network of the studied genes (represented by the circles in the inner ring; *HDAC4, TCF3, FGFR1, PIK3R2, MAP3K10, DNMT1, NPAT, ATM, BCL2L11, SKI, AGRN, MYO1C*) generated by automatically selected weighting method using GeneMANIA Application version: 3.5.0 A colour code in each of the circle (gene) indicates the associated molecular function.

As stated earlier, the genes for the present were selected based on their importance in several cellular pathways including DDR response and epigenetic regulation, among others. Taken together changes in methylation and/or expression of the selected genes, specifically by MWCNT, would be expected to influence multitude of cellular functions (Figure 6) including “repressing transcription factor binding”, “DNA methylation”, “intrinsic apoptotic signalling pathway” and “chromatin remodelling”. In addition to the genes reported in the present study, associated genes observed from the network (outer ring in Figure 6) included DNMT3B, several members of HDAC (HDAC1, HDAC3, HDAC5, and HDAC9) and Bcl-2 family; which have also been implicated in several diseases including lung cancer. And thus, epigenetic changes and changes in expression of the genes reported in the present study could be expected to have a cascade- effect leading to dysregulation of signalling pathway and cellular response.

In addition to changes in DNA methylation, studying miRNA expression often provides key information on the dysregulation of cellular processes. With several identified human miRNAs and their established role in gene regulation, especially in cancer [72, 73] and lung cancer [74]; we investigated changes in miRNA expression in cells exposed to CNTs. We did not observe any significant alteration in the miRNA profile at fold change cut-off of > 2 and FDR-corrected p values of < 0.05. Though not significant, some miRNAs, such as hsa-miR-601 was upregulated in MWCNT exposure (Relative Quantification, *RQ* = 1306.83) while it was downregulated by SWCNT (*RQ* = 0.01). Since, it has been reported that hsa-miR-601 can down-regulate Fas-induced apoptosis pathway and repressing nuclear factor-kappaB (*NF-*κ*B*) transcription factor in A549 cells [75], it could be of interest. Another miRNA, which was up regulated by SWCNT was hsa-mir-34b (*RQ* = 1.98), but the increase was not significant. Interestingly miR-34 family is associated with p53 network and is believed to be involved in some forms of cancers, and can be correlated with poor survival in smokers suffering from squamous cell carcinoma [74]. However, since the levels of miRNA were not significantly altered and was measured only after 24 h of exposure it is difficult to comment on the biological implication.

In summary, while the genes studied are not representative of methylation pattern of the whole genome, changes in their methylation and expression certainly provides important evidence of dysregulation of genes involved in critical pathways. MWCNT induced more changes in expression and/or DNA methylation of the studied genes, when compared to SWCNT. No significant changes in miRNA expression were observed for both MWCNT and SWCNT. Given the dynamic nature of these changes (DNA methylation, expression and miRNA), and since all the endpoints were studied at a single time point, it might be important to further study these changes over different time points after exposure.

## MATERIALS AND METHODS

### Nanomaterial characterization

Reference materials were obtained from European Commission JRC (MWCNT; JRC-NM400) and US NIST (SWCNT; NIST-SRM2483). The particles were characterized using TEM for primary size [28]. The protocol from European Union project Engineered Nanoparticle Risk Assessment (ENPRA) was used to prepare dispersion as described earlier [28]. DLS measurements were performed for the different concentrations in the cell culture media, at 1 and 24 h.

### Study design and exposure condition

Dr. Gruenert (University of California, San Francisco) provided 16 HBE human bronchial epithelial cell lines. The cells were cultured in DMEM/F12 supplemented with 5% of FCS and 1% of Penicillin-Streptomycin (10000 U/ml), L-glutamine (200 mM) and, Amphotericin-B (250 µg/ml), incubated at 37°C in a 100% humidified atmosphere containing 5% CO_2_. Cell culture medium was renewed every 2 days and sub-cultured at confluence. Stock of cells was generated and kept in liquid nitrogen to avoid the effect of cell stock/source. Cells from the same stock were grown until passage 4 for epigenetic experiments to avoid influence of cell passage/aging on epigenetic changes. Cells were seeded in wells of plates at density of 2 × 10^5^ cells/cm^2^ and allowed to attach for 24 h to reach 80% confluence and were exposed for 24 h to MWCNT and SWCNT. Cells were exposed in duplicates per condition, for three independent experiments.

Our previous experiments, *in vitro* (in THP-1 cells) [28], *in vivo* [27] and in human [17] formed the basis of the present study. For the present study, an exposure duration of 24 h was selected, so that the results could be comparable to that available data on cyto- and genotoxicity assays. Exposure concentrations (2.5, 5 and 25 μg/ml) were selected based on our preliminary cyto-genotoxicity screening [28]. Global and sequence specific methylation were studied in all the concentrations (2.5, 5 and 25 μg/ml), while miRNA expression and RNA expression were studied for a single concentration (25 μg/ml). Each sample was done in triplicates. Decitabine (1 μM) was used as a positive control for the experiment. DNA, RNA and miRNA were extracted using AllPrep DNA/RNA/miRNA Universal Kit (QIAGEN, Belgium). At the onset, global DNA hydroxymethylation was evaluated. Subsequently, a set of twelve differentially expressed genes were selected from a RNA-sequencing (RNA-seq) data. The differentially expressed genes were also studied for sequence-specific methylation changes. Additionally, miRNA expression was studied in the exposed cells.

### Global DNA methylation-LC/MS/MS

Global DNA methylation and hydroxymethylation was evaluated using LC-MS/MS based method described earlier [76, 77], that allows simultaneous detection of 2′-deoxycytidine (dC), 5-methyl-2′-deoxycytidine (5-mC), and 5-hydroxymethyl-2´-deoxycytidine (5-hmC). The results were expressed as % DNA methylation calculated as 5-mdC/(5-mdC + 5-OHmdC + dC), and % DNA hydroxy-methylation, calculated as 5-OHmdC/(5-mdC + 5-OHmdC + dC).

### RNA expression and sequence specific methylation

Results of differential expression of the genes used for pyrosequencing was extracted from the data set obtained from RNA sequencing. RNA sequencing was performed on a HiSeq2000 (Illumina) as previously described [78]. Briefly, RNA libraries were created using the Illumina TruSeq RNA sample preparation kit V2 according to the manufacturer’s instructions and sequencing reads were mapped to the human transcriptome and reference genome (GRCh37.65/hg19) by using TopHat 2.0 14 and Bowtie 2.0. Differential expression between the different exposures was calculated by using EdgeR16. Detailed analysis of the RNA sequencing dataset will be published elsewhere after detailed analysis of gene ontology, pathway analysis and gene functional classification analysis. For the present study however, data for 12 functionally important genes were used to investigate association with sequence specific methylation.

Sequence specific methylation of repetitive element (*LINE-1*) and gene promoters (*SKI, DNMT 1, HDAC 4, NPAT/ATM, BCL2L11, MAP3K10, PIK3R2, MYO1C, TCF3, FGFR 1, AGRN*) were analyzed using bisulfite pyrosequencing, as described previously [34]. In addition to RNA sequencing results, we obtained the present set of genes based on our preliminary experiments *in vitro* (in THP-1 cells, 16 HBE) [28, 29], *in vivo* [27] and human [17]. The set of genes for the present study have critical function in the pathways associated with DNA damage repair response. Bisulfite converted genomic DNA was amplified using Qiagen proprietary PCR primers (Supplementary Table 1, additional sequence information in Supplementary Figure 1), subsequently immobilized onto streptavidin sepharose beads and sequenced using the PyroMark Q24 (Qiagen). The results were analyzed using the PyroMark analysis 2.0.7 software (Build 3, Qiagen). Technical variability six samples (3-negative control and 3- positive control) were selected for technical variation analysis for some of the randomly selected genes and have been provided in the supplementary file (Supplementary Figure 2).

### miRNA expression

MiRNA profiling was performed with the TaqMan^®^ Array Human MicroRNA panel. Briefly, extracted RNA was reverse transcribed, followed by pre-amplification. The pre-amplified products were loaded onto the TaqMan^®^ panel and run on the QuantStudio 12K Flex system. Data analysis was performed using DataAssist v3.01. We selected only those samples with detectable Ct values for all the replicates/concentrations, with average Ct values < 35. Global normalization was performed on the data set. Mean relative quantity (RQ) was calculated and miRNAs differentially expressed between groups were defined as those with > 2- fold change and *P <* 0.05 (two tailed Student’s *t*-test), followed by adjustment using the false discovery rate (FDR) correction.

### Statistical analysis

For the results of global DNA methylation and hydroxymethylation and sequence-specific methylation, statistical analysis was performed using GraphPad Prism 5.0 (GraphPad Software, Inc.). Statistical significance using two-tailed unpaired Student’s *t*-test with *P <* 0.05 was considered significant.

## SUPPLEMENTARY MATERIALS FIGURES AND TABLES






