# Supplementary material for: Single-walled and multi-walled carbon nanotubes induce sequence-specific epigenetic alterations in 16 HBE cells
Source: Oncotarget. 2018 Apr 17;9(29):20351–65. doi: 10.18632/oncotarget.24866 (PMC5945544; doi:10.18632/oncotarget.24866)
Supplement: Supplementary file 1 [file oncotarget-09-20351-s001.pdf]

Single-walled and multi-walled carbon nanotubes induce sequence-specific epigenetic alterations in 16 HBE cells

SUPPLEMENTARY MATERIALS

Supplementary Table 1: Summary of primers used for the pyrosequencing assays

Gene Symbol	Gene name	Entrez Gene ID	Amplicon length	Sequence analysed	Qiagen product code
DNMT 1	DNA (cytosine-5-)-methyltransferase 1	1786	196 bp	**C**GCATG**C**GCAAGG**C**GGGCACACACCCCCCCCA**C**G**C**GGA	PM00075761
HDAC 4	Histone deacetylase 4	9759	95 bp	GGCTAA**C**G**C**GGGGAGC**C**GGCCAGGCCT**C**GC**C**GCGG**C**GT	PM00007539
NPAT/ATM	Nuclear protein ataxia-telangiectasia locus	4863/472	203 bp	C**G**C**G**GAC**G**C**G**GGAWGGAGGGTTATTGGACCC**G**GC	PM00153622
PIK3R2	Phosphoinositide-3-kinase, regulatory subunit 2 (beta)	5296	241 bp	TC**G**GGTC**G**GCGAGGGATCCGA**G**ACTGGGCTC**G**GC	PM00188601
BCL2L11	BCL2-like 11 (apoptosis facilitator)	10018	204 bp	C**G**C**G**GCTC**G**GCCC**G**GCTCTGGC**G**CTCTCTCC**G**CCC**G**T	PM00010759
MAP3K10	Mitogen-activated protein kinase kinase kinase 10	4294	260 bp	CC**C**GC**C**GA**C**GCC**C**GCAGCC**C**GCTGTTTGGGGGC**C**GC	PM00190022
AGRN	Agrin	375790	153 bp	GGGGTGTG**C**GGGGG**C**GCTGAGGTG**C**GGT**C**G**C**CGA	PM00094836
MYO1C	Myosin IC	4641	250 bp	GACCTCG**C**TGCCCCCACCCGC**C**GC**C**TTCCCT**C**GGC**C**GGA**C**GC	PM00069734
FGFR1	Fibroblast growth factor receptor 1	2260	219 bp	GCA**C**GTCCCAGAGGG**C**GC**C**GGA	PM00136003
TCF3	Transcription factor 3	6929	189 bp	**C**GGAAGC**C**G**C**GAAAGTCTGATGGG**C**GGGGA	PM00072219
SKI	SKI proto-oncogene	6497	126 bp	G**C**G**C**GCCT**C**GGC**C**GCTGCCTGGA**C**GA**C**GT	PM00004011

Supplementary Table 2: Results of pyrosequencing assays. See Supplementary_Table_2

Supplementary Table 3: Summary of miRNA expression changes induced by MWCNT and SWCNT. See Supplementary_Table_3

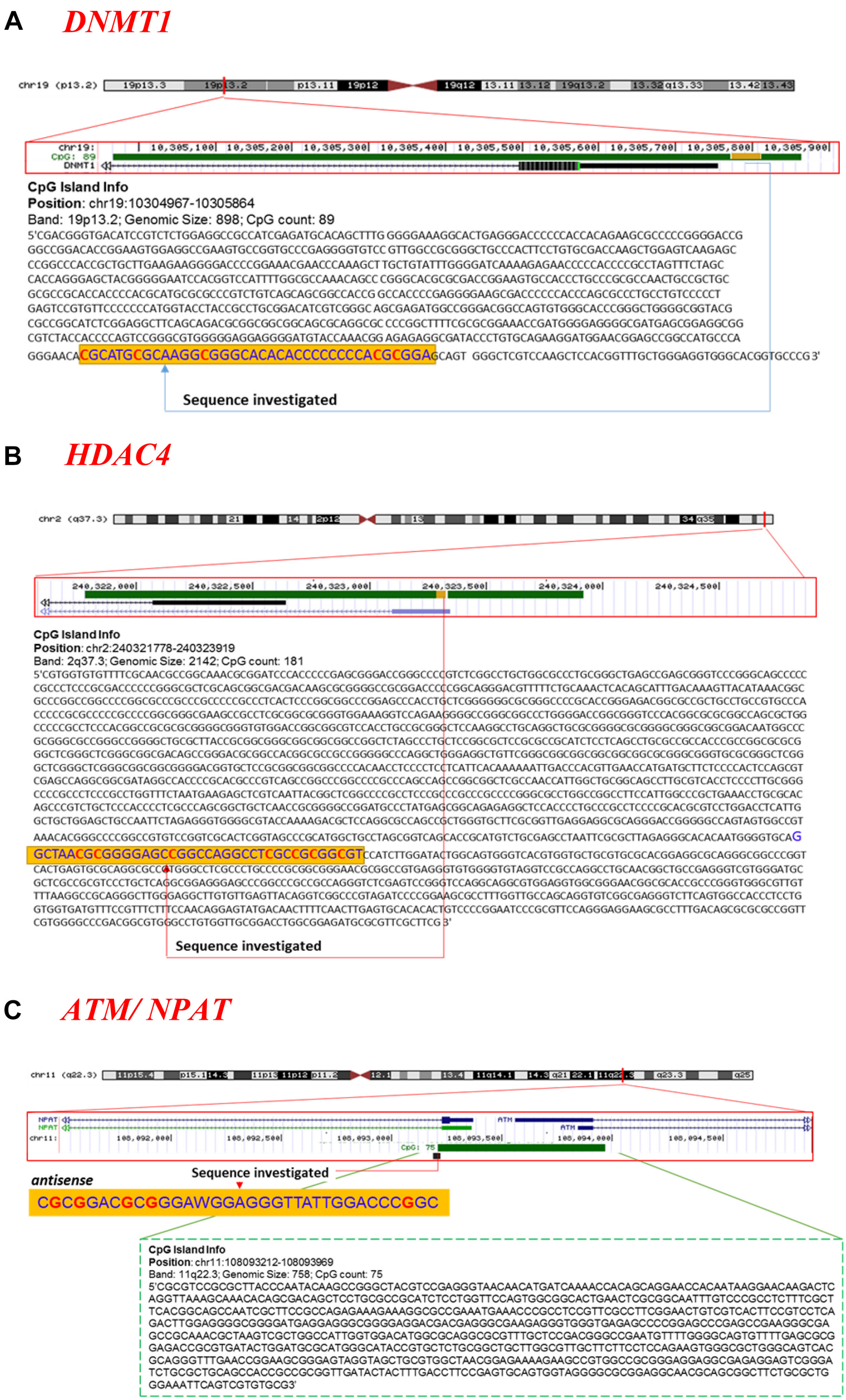
A DNMT1
chr19 (p13.2)
CpG: 89
DNMT1
CpG Island Info
Position: chr19:10304967-10305864
Band: 19p13.2; Genomic Size: 898; CpG count: 89
CGCATGCGCAAGGCGGGCACACACCCCCCCACGCGGA
Sequence investigated
B HDAC4
chr2 (q37.3)
CpG Island Info
Position: chr2:240321778-240323919
Band: 2q37.3; Genomic Size: 2142; CpG count: 181
GCTAACGCGGGGAGCCGGCCAGGCCTCGCCGCGGCGT
Sequence investigated
C ATM/ NPAT
chr11 (q22.3)
NPAT
ATM
CpG: 75
Sequence investigated
antisense
CGCGGACGCGGGAWGGAGGGTTATTGGACCCGGC
CpG Island Info
Position: chr11:108093212-108093969
Band: 11q22.3; Genomic Size: 758; CpG count: 75

D ***PIK3R2***

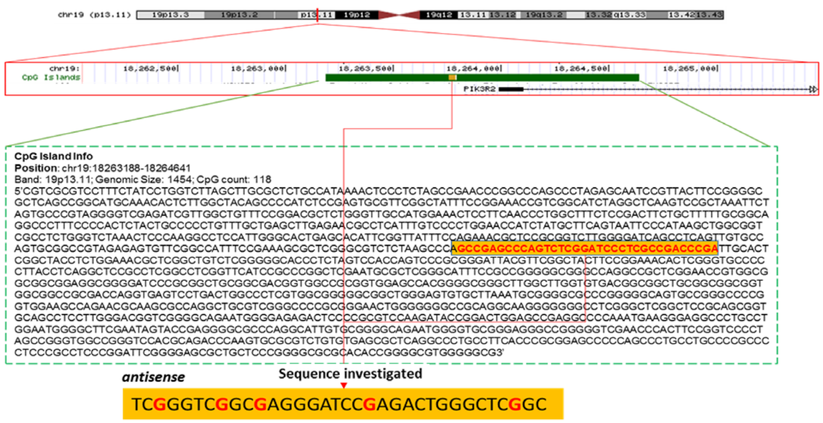

E ***BCL2L11***

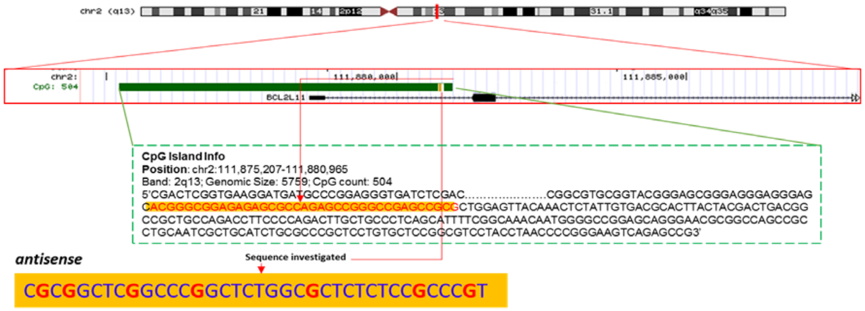

F ***MAP3K10***

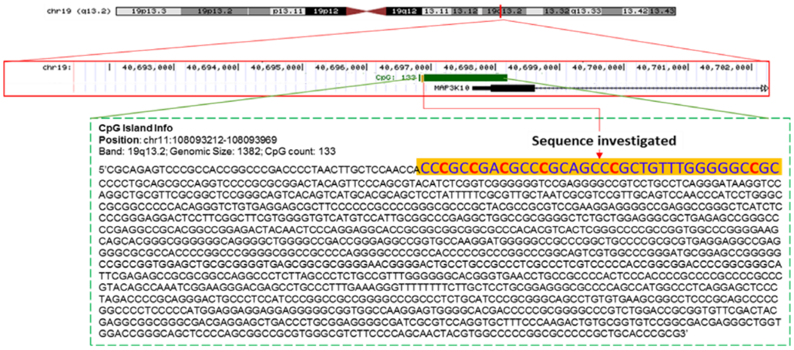

G ***AGRN***

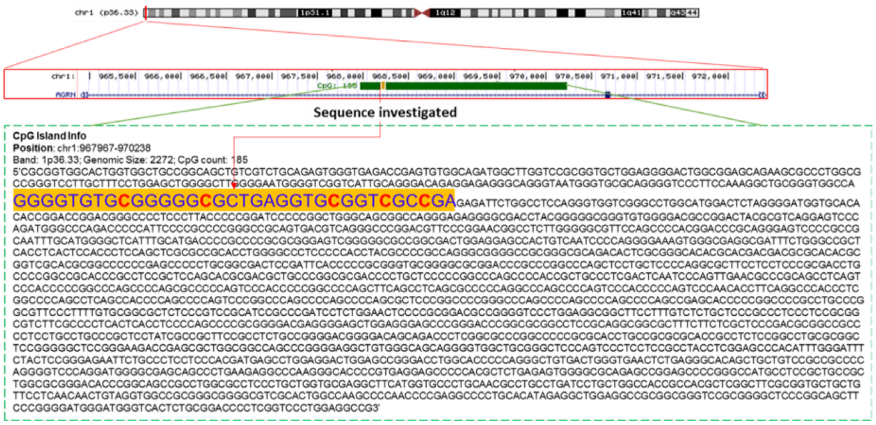

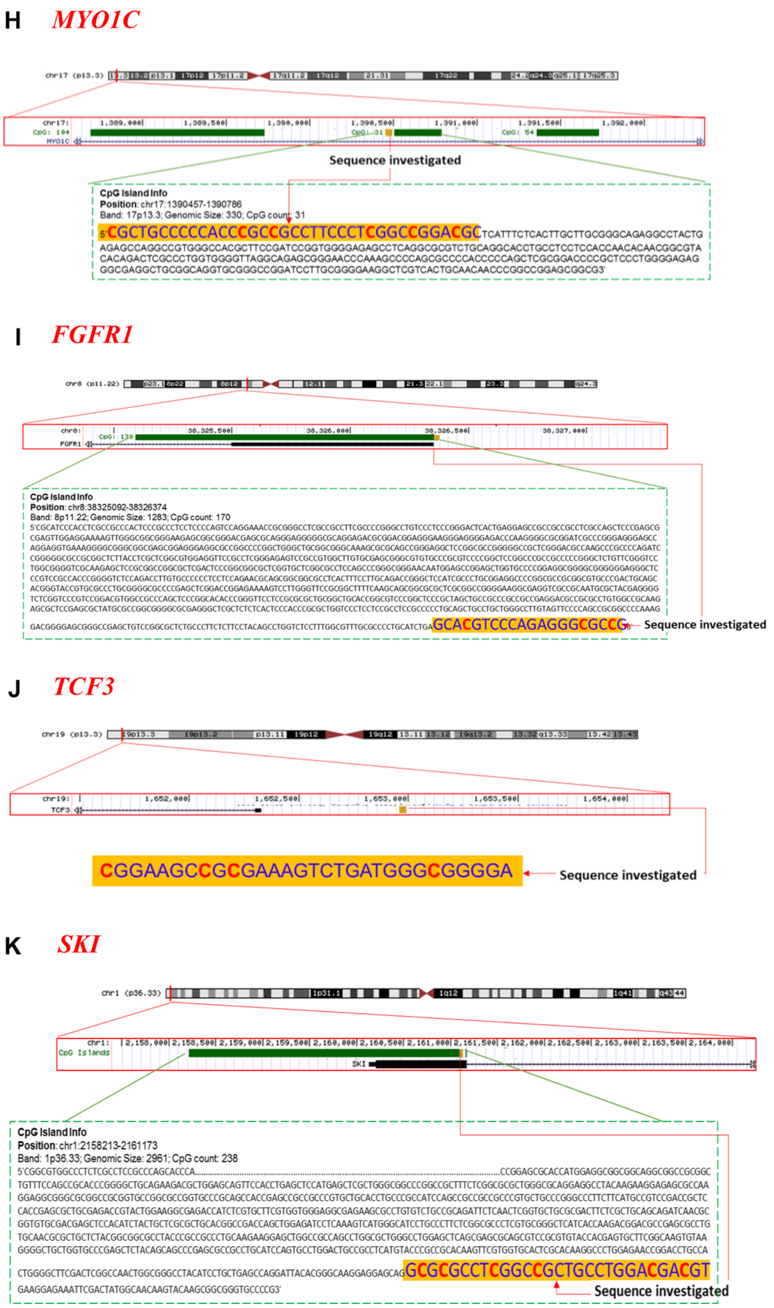

Supplementary Figure 1: Schematic representation showing the chromosome position, location of the CpG site within CpG island (where applicable) and the sequence region used for analysis of methylation for (**A**) *DNMT1* (**B**) *HDAC4* (**C**) *NPAT/ATM* (**D**) *PIK3R2* (**E**) *BCL2L11* (**F**) *MAP3K10* (**G**) *AGRN* (**H**) *MYO1C* (**I**) *FGFR1* (**J**) *TCF3* and (**K**) *SKI*. CpG islands are indicated by the green bar, with the highlighted region in yellow showing the sequence analysed, CpG sites are indicated in red coloured font.

Investigation of technical variability for pyrosequencing:

Six samples (3-negative control and 3- positive control) were selected for technical variation analysis. The samples were studied for methylation of LINE-1 elements, DNMT1 and HDAC4. Some of the results are presented below. The differences between the tested samples were not statistically significant usually with an error percentage of less than 5%.

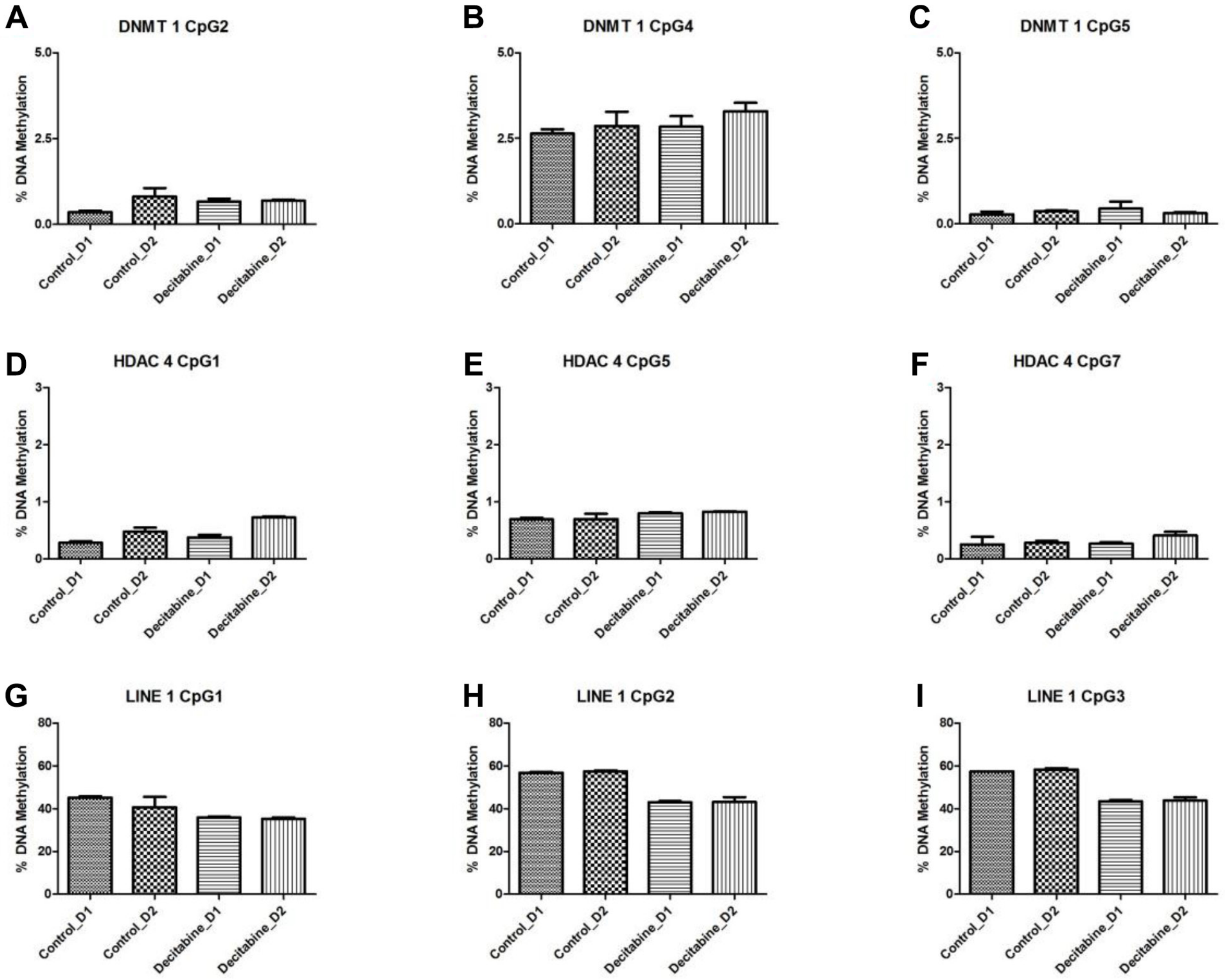

Supplementary Figure 2: Results showing changes in sequence specific methylation of (**A**) *DNMT1* CpG2, (**B**) *DNMT1* CpG4 (**C**) *DNMT1* CpG5 (**D**) *HDAC4* CpG1, (**E**) *HDAC4* CpG5, (**F**) *HDAC4* CpG7, (**G**) *LINE-1* CpG1, (**H**) *LINE-1* CpG2, (**I**) *LINE-1* CpG3, negative control (Control, $n = 3$) and positive control (Decitabine, $n = 3$) samples were analysed on two different occasions represented by the suffix '_D1' and '_D2'.

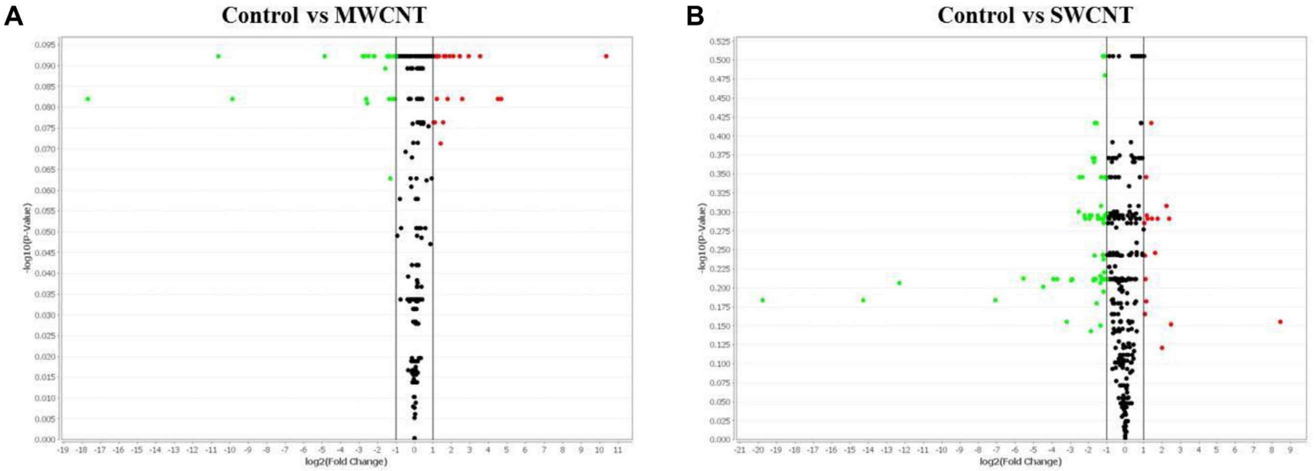

Supplementary Figure 3: Volcano plot analysis of differentially expressed miRNAs in cells exposed to (**A**) MWCNT and (**B**) SWCNT, the difference in miRNA expression is plotted on the x axis (log^{2} scale), and false discovery rate (FDR)-*p* value is plotted on the y axis (–log^{10} scale). Upregulated and downregulated miRNAs are indicated in red and green, respectively.
