# Supplementary material for: Single-walled and multi-walled carbon nanotubes induce sequence-specific epigenetic alterations in 16 HBE cells
Source: Oncotarget. 2018 Apr 17;9(29):20351–65. doi: 10.18632/oncotarget.24866 (PMC5945544; doi:10.18632/oncotarget.24866)
Supplement: Supplementary file 2 [file oncotarget-09-20351-s002.docx]

**Supplementary Table 3**: Summary of miRNA expression changes induced by MWCNT and SWCNT

|  | **Control Vs MWCNT** | | **Control Vs SWCNT** | |
| --- | --- | --- | --- | --- |
|  | **RQ** | **FDR adjusted p-value** | **RQ** | **FDR adjusted p-value** |
| 002102_hsa-miR-34b_B | 1.29 | 0.81 | 1.98 | 0.31 |
| 002277_hsa-miR-320_A | 1.21 | 0.81 | 1.57 | 0.31 |
| 002186_hsa-miR-345_A | 1.16 | 0.81 | 1.56 | 0.31 |
| 002276_hsa-miR-222_A | 1.12 | 0.81 | 1.45 | 0.31 |
| 002299_hsa-miR-191_A | 1.07 | 0.81 | 1.43 | 0.31 |
| 002187_hsa-miR-942_B | 1.18 | 0.81 | 1.31 | 0.31 |
| 002758_HSA-MIR-1226#_B | 0.52 | 0.81 | 0.63 | 0.31 |
| 000515_hsa-miR-212_A | 0.89 | 0.81 | 0.44 | 0.31 |
| 001510_hsa-miR-656_B | 1.17 | 0.81 | 0.43 | 0.31 |
| 000382_hsa-let-7f_A | 0.92 | 0.81 | 0.79 | 0.31 |
| 002844_HSA-MIR-320B_B | 0.90 | 0.86 | 0.46 | 0.31 |
| 002285_hsa-miR-186_A | 1.11 | 0.92 | 2.06 | 0.31 |
| 002260_hsa-miR-342-3p_A | 0.95 | 0.93 | 1.82 | 0.31 |
| 002840_HSA-MIR-1275_B | 1.05 | 0.93 | 0.55 | 0.31 |
| 001821_hsa-miR-484_A | 1.04 | 0.96 | 1.68 | 0.31 |
| 002160_hsa-miR-148b#_B | 2.45 | 0.81 | 2.68 | 0.38 |
| 001606_hsa-miR-661_B | 0.83 | 0.81 | 0.32 | 0.38 |
| 002289_hsa-miR-139-5p_A | 1.02 | 0.96 | 1.80 | 0.38 |
| 002302_hsa-miR-425#_B | 0.97 | 0.97 | 1.81 | 0.38 |
| 002316_hsa-miR-34a#_B | 1.28 | 0.81 | 1.24 | 0.41 |
| 002295_hsa-miR-223_A | 0.54 | 0.81 | 0.62 | 0.41 |
| 000419_hsa-miR-30c_A | 1.22 | 0.81 | 1.27 | 0.42 |
| 001094_RNU44_B | 1.07 | 0.81 | 0.81 | 0.42 |
| 000497_hsa-miR-197_A | 1.26 | 0.81 | 1.92 | 0.43 |
| 002240_hsa-miR-542-5p_A | 1.31 | 0.81 | 1.80 | 0.43 |
| 002897_HSA-MIR-664_B | 1.57 | 0.81 | 1.77 | 0.43 |
| 002432_hsa-miR-625#_B | 1.40 | 0.81 | 1.67 | 0.43 |
| 002677_HSA-MIR-590-3P_B | 1.36 | 0.81 | 1.67 | 0.43 |
| 002445_hsa-miR-27a#_B | 1.91 | 0.81 | 1.42 | 0.43 |
| 001338_rno-miR-7#_B | 1.16 | 0.81 | 1.35 | 0.43 |
| 002325_hsa-miR-744#_B | 1.21 | 0.81 | 0.62 | 0.43 |
| 000539_hsa-miR-324-5p_A | 1.23 | 0.81 | 0.61 | 0.43 |
| 002895_HSA-MIR-720_B | 0.77 | 0.81 | 0.54 | 0.43 |
| 002883_HSA-MIR-1274A_B | 0.65 | 0.81 | 0.31 | 0.43 |
| 002884_HSA-MIR-1274B_B | 0.69 | 0.81 | 0.31 | 0.43 |
| 001284_hsa-miR-542-3p_A | 0.66 | 0.81 | 0.29 | 0.43 |
| 000527_hsa-miR-296_A | 1.10 | 0.83 | 0.72 | 0.43 |
| 002184_hsa-miR-339-3p_A | 0.95 | 0.85 | 1.44 | 0.43 |
| 000437_hsa-miR-100_A | 1.04 | 0.88 | 0.69 | 0.43 |
| 002159_hsa-miR-135b#_B | 0.96 | 0.93 | 0.54 | 0.43 |
| 000580_hsa-miR-20a_A | 1.02 | 0.96 | 0.72 | 0.43 |
| 001141_mmu-miR-451_A | 1.05 | 0.96 | 0.30 | 0.43 |
| 002255_hsa-miR-149_A | 1.15 | 0.81 | 1.41 | 0.43 |
| 000491_hsa-miR-192_A | 1.14 | 0.81 | 1.34 | 0.43 |
| 002908_HSA-MIR-1296_B | 0.70 | 0.81 | 0.60 | 0.43 |
| 002142_hsa-miR-100#_B | 0.47 | 0.81 | 0.31 | 0.43 |
| 001551_hsa-miR-597_A | 2.46 | 0.81 | 2.18 | 0.45 |
| 002870_HSA-MIR-1248_B | 1.63 | 0.81 | 0.20 | 0.45 |
| 002819_HSA-MIR-548K_B | 3.01 | 0.81 | 0.18 | 0.45 |
| 002301_hsa-miR-22#_B | 0.93 | 0.84 | 0.69 | 0.45 |
| 000560_hsa-miR-372_A | 0.90 | 0.91 | 1.73 | 0.45 |
| 000545_hsa-miR-331_A | 1.07 | 0.91 | 0.68 | 0.45 |
| 000449_hsa-miR-125b_A | 1.07 | 0.93 | 0.47 | 0.45 |
| 000403_hsa-miR-25_A | 1.03 | 0.94 | 0.78 | 0.45 |
| 002174_hsa-miR-27b#_B | 0.95 | 0.96 | 0.59 | 0.45 |
| 001611_hsa-miR-654_A | 1.03 | 0.96 | 0.55 | 0.45 |
| 002438_hsa-miR-21#_B | 0.85 | 0.83 | 0.49 | 0.45 |
| 002367_hsa-miR-193b_A | 1.09 | 0.81 | 1.16 | 0.46 |
| 001996_hsa-miR-454#_B | 1.64 | 0.81 | 4.73 | 0.49 |
| 002139_hsa-miR-93#_B | 1.15 | 0.81 | 1.65 | 0.49 |
| 000391_hsa-miR-16_A | 1.21 | 0.81 | 1.19 | 0.49 |
| 000489_hsa-miR-190_A | 1.14 | 0.91 | 0.40 | 0.49 |
| 000602_hsa-miR-30b_A | 1.23 | 0.81 | 1.17 | 0.50 |
| 000452_hsa-miR-127_A | 1.25 | 0.81 | 0.72 | 0.50 |
| 000420_hsa-miR-30d_B | 1.21 | 0.81 | 0.65 | 0.50 |
| 001285_hsa-miR-487b_A | 1.54 | 0.81 | 1.51 | 0.50 |
| 000426_hsa-miR-34a_A | 1.20 | 0.81 | 0.58 | 0.50 |
| 002436_hsa-miR-629_A | 1.24 | 0.83 | 0.61 | 0.50 |
| 000454_hsa-miR-130a_A | 1.15 | 0.84 | 0.50 | 0.50 |
| 000475_hsa-miR-152_A | 1.07 | 0.87 | 0.72 | 0.50 |
| 000439_hsa-miR-103_A | 1.02 | 0.96 | 0.58 | 0.50 |
| 000456_hsa-miR-130b_A | 0.99 | 0.98 | 0.54 | 0.50 |
| 002446_hsa-miR-28-3p_A | 0.88 | 0.81 | 1.31 | 0.51 |
| 002419_hsa-miR-15a#_B | 1.50 | 0.81 | 2.24 | 0.51 |
| 002227_hsa-miR-323-3p_A | 1.26 | 0.81 | 1.36 | 0.51 |
| 002279_hsa-miR-31_A | 1.26 | 0.81 | 1.35 | 0.51 |
| 001984_hsa-miR-590-5p_A | 1.52 | 0.81 | 1.09 | 0.51 |
| 002308_hsa-miR-17_A | 1.18 | 0.81 | 0.90 | 0.51 |
| 001986_hsa-miR-766_B | 0.88 | 0.81 | 0.83 | 0.51 |
| 000546_hsa-miR-335_A | 1.31 | 0.81 | 0.81 | 0.51 |
| 001998_hsa-miR-769-5p_B | 0.87 | 0.81 | 0.77 | 0.51 |
| 000417_hsa-miR-30a-5p_B | 1.17 | 0.81 | 0.71 | 0.51 |
| 002441_hsa-miR-24-2#_B | 0.67 | 0.81 | 0.58 | 0.51 |
| 000480_hsa-miR-181a_A | 1.37 | 0.81 | 0.58 | 0.51 |
| 002231_hsa-miR-9#_B | 1.36 | 0.81 | 0.54 | 0.51 |
| 001582_hsa-miR-638_B | 0.62 | 0.81 | 0.52 | 0.51 |
| 001535_hsa-miR-551b_A | 1.34 | 0.81 | 0.47 | 0.51 |
| 002434_hsa-miR-628-3p_B | 0.15 | 0.81 | 0.26 | 0.51 |
| 002818_HSA-MIR-1254_B | 0.59 | 0.81 | 0.22 | 0.51 |
| 001612_hsa-miR-655_A | 0.88 | 0.81 | 0.69 | 0.51 |
| 000583_hsa-miR-9_A | 1.14 | 0.83 | 0.62 | 0.51 |
| 002422_hsa-miR-18a_A | 1.15 | 0.83 | 0.55 | 0.51 |
| 002392_hsa-miR-301b_A | 1.06 | 0.89 | 0.72 | 0.51 |
| 001516_hsa-miR-425-5p_A | 1.10 | 0.92 | 1.35 | 0.51 |
| 002352_hsa-miR-652_A | 0.92 | 0.93 | 0.51 | 0.51 |
| 001818_rno-miR-29c#_B | 1.17 | 0.96 | 0.35 | 0.51 |
| 000399_hsa-miR-23a_A | 1.03 | 0.96 | 0.66 | 0.51 |
| 002779_HSA-MIR-1271_B | 2.18 | 0.81 | 5.29 | 0.51 |
| 002322_hsa-miR-671-3p_A | 1.45 | 0.81 | 3.35 | 0.51 |
| 002323_hsa-miR-454_A | 1.38 | 0.81 | 2.73 | 0.51 |
| 002433_hsa-miR-628-5p_A | 2.27 | 0.81 | 2.34 | 0.51 |
| 002259_hsa-miR-340#_B | 1.21 | 0.81 | 1.25 | 0.51 |
| 002198_hsa-miR-125a-5p_A | 1.31 | 0.81 | 1.22 | 0.51 |
| 002202_hsa-miR-889_A | 1.49 | 0.81 | 1.20 | 0.51 |
| 000524_hsa-miR-221_A | 1.05 | 0.81 | 0.94 | 0.51 |
| 000436_hsa-miR-99b_A | 1.11 | 0.81 | 0.78 | 0.51 |
| 001039_hsa-miR-492_A | 0.64 | 0.81 | 0.39 | 0.51 |
| 002249_hsa-miR-143_A | 0.36 | 0.81 | 0.36 | 0.51 |
| 002863_HSA-MIR-1290_B | 0.46 | 0.81 | 0.26 | 0.51 |
| 002857_HSA-MIR-663B_B | 0.63 | 0.81 | 0.22 | 0.51 |
| 001562_hsa-miR-629_B | 1.09 | 0.89 | 1.51 | 0.51 |
| 002217_hsa-miR-18b_A | 1.13 | 0.91 | 0.44 | 0.51 |
| 002109_hsa-miR-32_A | 1.10 | 0.93 | 1.73 | 0.51 |
| 000398_hsa-miR-22_A | 1.05 | 0.96 | 0.67 | 0.51 |
| 002281_hsa-miR-193a-5p_A | 0.94 | 0.96 | 0.55 | 0.51 |
| 002215_hsa-miR-196b_A | 1.04 | 0.96 | 1.50 | 0.51 |
| 001011_hsa-miR-200a#_B | 1.76 | 0.81 | 2.06 | 0.52 |
| 002619_hsa-let-7b_A | 1.47 | 0.81 | 1.48 | 0.52 |
| 000389_hsa-miR-15a_A | 1.70 | 0.81 | 1.24 | 0.52 |
| 000402_hsa-miR-24_A | 1.24 | 0.81 | 1.08 | 0.52 |
| 002792_HSA-MIR-1303_B | 0.69 | 0.81 | 0.61 | 0.52 |
| 002165_hsa-miR-29b-1#_B | 1.31 | 0.81 | 0.60 | 0.52 |
| 002380_hsa-miR-106b#_B | 0.85 | 0.87 | 0.52 | 0.52 |
| 001280_hsa-miR-455_A | 1.32 | 0.81 | 0.71 | 0.53 |
| 002117_hsa-miR-362-3p_A | 1.80 | 0.81 | 2.00 | 0.53 |
| 000379_hsa-let-7c_A | 1.06 | 0.96 | 1.52 | 0.55 |
| 002185_hsa-miR-335#_B | 1.46 | 0.81 | 1.55 | 0.57 |
| 001610_hsa-miR-411_A | 1.50 | 0.81 | 1.26 | 0.57 |
| 000572_hsa-miR-382_A | 1.58 | 0.81 | 0.72 | 0.57 |
| 000413_hsa-miR-29b_A | 1.26 | 0.81 | 0.56 | 0.57 |
| 002447_hsa-miR-29a#_B | 1.09 | 0.92 | 0.63 | 0.57 |
| 002218_hsa-miR-10b_A | 0.84 | 0.93 | 3.09 | 0.57 |
| 000387_hsa-miR-10a_A | 1.05 | 0.94 | 0.73 | 0.57 |
| 000508_hsa-miR-204_A | 1.19 | 0.93 | 1.89 | 0.57 |
| 001973_U6 rRNA_B | 1.15 | 0.81 | 1.16 | 0.57 |
| 000442_hsa-miR-106b_A | 1.27 | 0.81 | 0.74 | 0.57 |
| 002340_hsa-miR-423-5p_A | 1.21 | 0.81 | 0.71 | 0.57 |
| 000544_hsa-miR-330_A | 1.37 | 0.81 | 0.63 | 0.57 |
| 002904_HSA-MIR-548L_B | 2.00 | 0.81 | 0.58 | 0.57 |
| 001583_hsa-miR-639_B | 0.63 | 0.81 | 0.43 | 0.57 |
| 000564_hsa-miR-375_A | 1.24 | 0.81 | 1.54 | 0.57 |
| 002317_hsa-miR-181a-2#_B | 1.29 | 0.81 | 1.21 | 0.57 |
| 002355_hsa-miR-532-3p_A | 1.33 | 0.81 | 1.19 | 0.57 |
| 002407_hsa-let-7e#_B | 2.13 | 0.84 | 1.91 | 0.57 |
| 000509_hsa-miR-205_A | 1.07 | 0.89 | 0.79 | 0.57 |
| 000482_hsa-miR-181c_A | 1.17 | 0.89 | 0.57 | 0.57 |
| 001048_hsa-miR-503_A | 1.11 | 0.93 | 0.51 | 0.57 |
| 002114_hsa-miR-130b#_B | 2.12 | 0.81 | 2.11 | 0.57 |
| 000507_hsa-miR-203_A | 1.20 | 0.81 | 0.87 | 0.57 |
| 002423_hsa-miR-18a#_B | 0.97 | 0.97 | 0.82 | 0.57 |
| 000561_hsa-miR-373_A | 0.88 | 0.96 | 0.44 | 0.58 |
| 002425_hsa-miR-19b-1#_B | 0.73 | 0.81 | 0.68 | 0.59 |
| 002244_hsa-miR-455-3p_A | 1.31 | 0.84 | 0.59 | 0.60 |
| 002257_hsa-miR-339-5p_A | 1.37 | 0.81 | 1.13 | 0.61 |
| 001182_mmu-miR-124a_A | 0.72 | 0.81 | 0.66 | 0.61 |
| 002442_hsa-miR-25#_B | 1.21 | 0.84 | 0.70 | 0.61 |
| 002233_hsa-miR-331-5p_A | 1.89 | 0.87 | 0.02 | 0.61 |
| 000554_hsa-miR-361_A | 1.06 | 0.93 | 0.89 | 0.61 |
| 002366_hsa-miR-193b#_B | 1.33 | 0.81 | 1.49 | 0.61 |
| 001187_mmu-miR-140_A | 1.32 | 0.81 | 1.26 | 0.61 |
| 001605_hsa-miR-548d_A | 2.14 | 0.81 | 1.15 | 0.61 |
| 000512_hsa-miR-210_A | 1.33 | 0.81 | 1.14 | 0.61 |
| 001518_hsa-miR-532_A | 0.98 | 0.81 | 0.93 | 0.61 |
| 000494_hsa-miR-195_A | 1.28 | 0.81 | 0.86 | 0.61 |
| 000408_hsa-miR-27a_A | 1.37 | 0.81 | 0.80 | 0.61 |
| 002262_hsa-miR-147b_A | 1.40 | 0.81 | 0.76 | 0.61 |
| 000397_hsa-miR-21_A | 1.26 | 0.81 | 0.74 | 0.61 |
| 002296_hsa-miR-885-5p_A | 0.48 | 0.81 | 0.68 | 0.61 |
| 000493_hsa-miR-194_A | 1.37 | 0.81 | 0.60 | 0.61 |
| 002409_hsa-miR-589_A | 0.66 | 0.81 | 0.60 | 0.61 |
| 000473_hsa-miR-150_A | 11.56 | 0.81 | 0.31 | 0.61 |
| 001608_hsa-miR-449b_A | 0.15 | 0.81 | 0.08 | 0.61 |
| 002437_hsa-miR-20a#_B | 1.15 | 0.81 | 0.81 | 0.61 |
| 002324_hsa-miR-744_A | 1.16 | 0.81 | 0.80 | 0.61 |
| 000604_hsa-miR-424_A | 1.24 | 0.83 | 0.73 | 0.61 |
| 000516_hsa-miR-213_B | 0.81 | 0.83 | 0.72 | 0.61 |
| 000435_hsa-miR-99a_A | 1.15 | 0.84 | 0.74 | 0.61 |
| 001119_hsa-miR-520e_A | 0.60 | 0.89 | 0.13 | 0.61 |
| 001553_hsa-miR-622_B | 0.78 | 0.91 | 0.45 | 0.61 |
| 002309_hsa-miR-424#_B | 1.10 | 0.92 | 1.22 | 0.61 |
| 000587_hsa-miR-29c_A | 1.23 | 0.93 | 0.49 | 0.61 |
| 000409_hsa-miR-27b_A | 1.09 | 0.93 | 0.71 | 0.61 |
| 002429_hsa-miR-548c-5p_A | 1.18 | 0.93 | 0.41 | 0.61 |
| 002283_hsa-let-7d_A | 1.05 | 0.93 | 0.78 | 0.61 |
| 002408_hsa-miR-548b-5p_A | 1.13 | 0.94 | 0.58 | 0.61 |
| 002254_hsa-miR-151-3p_B | 0.93 | 0.96 | 1.44 | 0.61 |
| 002096_hsa-miR-221#_B | 0.93 | 0.96 | 0.61 | 0.61 |
| 002271_hsa-miR-185_A | 1.02 | 0.96 | 0.84 | 0.61 |
| 001823_hsa-miR-512-3p_A | 1.06 | 0.96 | 1.10 | 0.61 |
| 002286_hsa-miR-200c#_B | 0.92 | 0.96 | 0.33 | 0.61 |
| 001543_hsa-miR-589_B | 0.98 | 0.97 | 0.72 | 0.61 |
| 002338_hsa-miR-483-5p_A | 1.00 | 1.00 | 0.55 | 0.61 |
| 002169_hsa-miR-106a_A | 1.10 | 0.81 | 0.90 | 0.62 |
| 000390_hsa-miR-15b_A | 1.19 | 0.81 | 0.81 | 0.62 |
| 001138_mmu-miR-379_A | 1.39 | 0.81 | 0.75 | 0.62 |
| 002351_hsa-miR-576-3p_A | 1.13 | 0.81 | 0.88 | 0.62 |
| 002769_HSA-MIR-1227_B | 2.91 | 0.84 | 0.13 | 0.62 |
| 000577_hsa-miR-98_A | 0.94 | 0.94 | 0.78 | 0.62 |
| 001006_RNU48_B | 1.00 | 0.98 | 1.02 | 0.62 |
| 000460_hsa-miR-135a_A | 1.36 | 0.81 | 0.72 | 0.62 |
| 000557_hsa-miR-369-3p_A | 0.37 | 0.81 | 0.39 | 0.62 |
| 001014_hsa-miR-20b_A | 1.06 | 0.93 | 0.79 | 0.62 |
| 000407_hsa-miR-26b_A | 1.25 | 0.81 | 0.86 | 0.63 |
| 000400_hsa-miR-23b_A | 0.03 | 0.81 | 0.05 | 0.63 |
| 002112_hsa-miR-29a_A | 1.20 | 0.81 | 0.81 | 0.63 |
| 002376_hsa-miR-543_B | 0.70 | 0.81 | 0.91 | 0.64 |
| 001286_hsa-miR-539_A | 1.02 | 0.97 | 0.88 | 0.64 |
| 002415_hsa-miR-519a_A | 1.00 | 1.00 | 0.44 | 0.64 |
| 002428_hsa-miR-500_A | 1.23 | 0.81 | 1.08 | 0.64 |
| 002297_hsa-miR-422a_A | 0.94 | 0.96 | 0.62 | 0.65 |
| 002413_hsa-miR-522_A | 5.40 | 0.81 | 0.60 | 0.66 |
| 001558_hsa-miR-601_B | 1306.83 | 0.81 | 0.01 | 0.66 |
| 002263_hsa-miR-190b_B | 0.16 | 0.83 | 0.00 | 0.66 |
| 002136_hsa-miR-33a#_B | 0.95 | 0.93 | 1.49 | 0.66 |
| 002161_hsa-miR-324-3p_A | 1.20 | 0.81 | 1.07 | 0.66 |
| 001319_mmu-miR-374-5p_A | 1.17 | 0.81 | 0.85 | 0.66 |
| 000443_hsa-miR-107_A | 1.10 | 0.93 | 0.76 | 0.66 |
| 002838_HSA-MIR-1291_B | 0.87 | 0.93 | 0.67 | 0.66 |
| 002199_hsa-miR-125a-3p_A | 1.05 | 0.96 | 1.42 | 0.66 |
| 000469_hsa-miR-147_A | 1.03 | 0.97 | 0.63 | 0.66 |
| 000521_hsa-miR-218_A | 1.31 | 0.81 | 0.87 | 0.67 |
| 002334_hsa-miR-182_A | 1.51 | 0.81 | 2.12 | 0.68 |
| 002274_hsa-miR-200b#_B | 0.86 | 0.93 | 0.66 | 0.68 |
| 000470_hsa-miR-148a_A | 1.12 | 0.93 | 0.76 | 0.68 |
| 002791_HSA-MIR-1244_B | 0.93 | 0.96 | 0.65 | 0.68 |
| 001043_hsa-miR-497_B | 0.96 | 0.97 | 0.60 | 0.68 |
| 000565_hsa-miR-376a_A | 1.32 | 0.81 | 1.13 | 0.70 |
| 002896_HSA-MIR-1260_B | 0.78 | 0.81 | 0.84 | 0.70 |
| 002122_hsa-miR-376c_A | 1.35 | 0.81 | 0.84 | 0.70 |
| 002305_hsa-miR-30d#_B | 1.56 | 0.81 | 0.84 | 0.70 |
| 002124_hsa-miR-371-3p_A | 0.40 | 0.81 | 0.63 | 0.70 |
| 001592_hsa-miR-642_A | 1.23 | 0.83 | 1.26 | 0.70 |
| 002781_HSA-MIR-1201_B | 1.24 | 0.83 | 1.18 | 0.70 |
| 001960_mmu-miR-615_A | 0.38 | 0.83 | 0.39 | 0.71 |
| 002089_hsa-miR-505_A | 1.20 | 0.81 | 0.78 | 0.71 |
| 000377_hsa-let-7a_A | 1.10 | 0.83 | 0.90 | 0.71 |
| 000518_hsa-miR-215_A | 0.86 | 0.81 | 1.11 | 0.72 |
| 001153_hsa-miR-517c_A | 0.68 | 0.81 | 0.81 | 0.72 |
| 000567_hsa-miR-378_B | 1.15 | 0.93 | 1.55 | 0.72 |
| 000428_hsa-miR-34c_A | 0.95 | 0.96 | 0.71 | 0.72 |
| 001020_hsa-miR-365_A | 1.01 | 0.99 | 0.78 | 0.74 |
| 001273_hsa-miR-362_A | 1.11 | 0.84 | 1.14 | 0.75 |
| 002349_hsa-miR-574-3p_A | 1.01 | 0.96 | 1.13 | 0.75 |
| 002083_hsa-miR-502-3p_A | 1.38 | 0.81 | 1.36 | 0.75 |
| 000431_hsa-miR-92a_A | 1.09 | 0.81 | 0.93 | 0.75 |
| 002270_hsa-miR-183#_B | 0.85 | 0.81 | 1.01 | 0.76 |
| 001186_mmu-miR-134_A | 0.93 | 0.96 | 1.19 | 0.76 |
| 000405_hsa-miR-26a_A | 1.11 | 0.81 | 0.93 | 0.76 |
| 002137_hsa-miR-92a-1#_B | 0.83 | 0.93 | 0.69 | 0.76 |
| 002400_hsa-miR-520c-3p_B | 0.94 | 0.98 | 3.97 | 0.76 |
| 002196_hsa-miR-99b#_B | 1.19 | 0.81 | 1.38 | 0.76 |
| 002333_hsa-miR-181c#_B | 1.52 | 0.81 | 1.05 | 0.77 |
| 001277_hsa-miR-485-3p_A | 1.75 | 0.81 | 1.29 | 0.77 |
| 002431_hsa-miR-625_A | 1.27 | 0.81 | 1.12 | 0.77 |
| 000563_hsa-miR-374_A | 1.36 | 0.81 | 0.93 | 0.77 |
| 002298_hsa-miR-129#_B | 1.33 | 0.84 | 0.83 | 0.77 |
| 001515_hsa-miR-660_A | 1.34 | 0.81 | 0.87 | 0.78 |
| 002420_hsa-miR-16-1#_B | 1.29 | 0.83 | 1.37 | 0.78 |
| 002173_hsa-miR-15b#_B | 1.13 | 0.84 | 0.94 | 0.79 |
| 001630_mmu-miR-491_A | 1.14 | 0.81 | 1.07 | 0.79 |
| 002087_hsa-miR-505#_B | 0.97 | 0.97 | 0.77 | 0.79 |
| 002331_hsa-miR-409-5p_A | 1.68 | 0.81 | 0.93 | 0.79 |
| 002100_hsa-miR-136#_B | 0.64 | 0.81 | 0.82 | 0.79 |
| 002801_HSA-MIR-1255B_B | 1.59 | 0.81 | 0.81 | 0.79 |
| 002177_hsa-miR-934_B | 2.16 | 0.81 | 0.68 | 0.79 |
| 002443_hsa-miR-26a-1#_B | 1.38 | 0.84 | 1.22 | 0.79 |
| 002095_hsa-miR-219-1-3p_A | 0.77 | 0.93 | 0.71 | 0.79 |
| 002182_hsa-miR-939_B | 1.37 | 0.84 | 0.85 | 0.79 |
| 001024_hsa-miR-429_A | 1.34 | 0.81 | 0.91 | 0.80 |
| 000528_hsa-miR-301_A | 1.13 | 0.85 | 0.91 | 0.80 |
| 000395_hsa-miR-19a_A | 1.30 | 0.81 | 1.08 | 0.81 |
| 002444_hsa-miR-26b#_B | 2.38 | 0.81 | 1.30 | 0.81 |
| 002847_HSA-MIR-1180_B | 0.99 | 0.99 | 1.19 | 0.82 |
| 001274_hsa-miR-410_A | 0.96 | 0.93 | 1.05 | 0.83 |
| 000502_hsa-miR-200a_A | 1.24 | 0.81 | 0.94 | 0.85 |
| 002237_hsa-miR-548d-5p_A | 1.75 | 0.81 | 0.86 | 0.85 |
| 001990_hsa-miR-758_A | 1.36 | 0.81 | 1.08 | 0.85 |
| 001101_hsa-miR-329_A | 1.07 | 0.96 | 0.83 | 0.85 |
| 002406_hsa-let-7e_A | 1.15 | 0.81 | 1.04 | 0.86 |
| 002398_hsa-miR-579_A | 1.66 | 0.81 | 1.07 | 0.87 |
| 002216_hsa-miR-128a_A | 1.13 | 0.88 | 0.93 | 0.87 |
| 001589_hsa-miR-616_B | 1.63 | 0.81 | 1.13 | 0.88 |
| 002284_hsa-miR-138_A | 1.44 | 0.81 | 1.11 | 0.88 |
| 002418_hsa-let-7f-2#_B | 1.90 | 0.81 | 0.90 | 0.88 |
| 002266_hsa-miR-545#_B | 0.58 | 0.88 | 0.78 | 0.88 |
| 000471_hsa-miR-148b_A | 1.05 | 0.96 | 1.07 | 0.88 |
| 002113_hsa-miR-31#_B | 1.36 | 0.81 | 0.95 | 0.88 |
| 002246_hsa-miR-133a_A | 0.70 | 0.81 | 0.92 | 0.89 |
| 000396_hsa-miR-19b_A | 1.29 | 0.81 | 1.04 | 0.90 |
| 002253_hsa-miR-101_A | 1.94 | 0.81 | 0.86 | 0.90 |
| 002384_hsa-miR-519b-3p_B | 1.13 | 0.96 | 1.28 | 0.90 |
| 002243_hsa-miR-378_B | 1.81 | 0.81 | 1.18 | 0.90 |
| 001663_mmu-miR-495_A | 1.19 | 0.81 | 0.96 | 0.90 |
| 002439_hsa-miR-23a#_B | 1.63 | 0.81 | 0.83 | 0.90 |
| 001097_hsa-miR-146b_A | 1.12 | 0.84 | 0.94 | 0.90 |
| 000543_hsa-miR-328_A | 0.96 | 0.96 | 0.96 | 0.90 |
| 002258_hsa-miR-340_A | 1.26 | 0.81 | 0.95 | 0.91 |
| 000463_hsa-miR-141_A | 1.54 | 0.81 | 1.04 | 0.91 |
| 002300_hsa-miR-200c_A | 1.24 | 0.89 | 0.95 | 0.91 |
| 002365_hsa-miR-494_A | 1.15 | 0.81 | 0.96 | 0.91 |
| 001090_mmu-miR-93_A | 1.26 | 0.81 | 0.99 | 0.92 |
| 002269_hsa-miR-183_A | 0.81 | 0.81 | 1.03 | 0.93 |
| 001109_hsa-miR-502_A | 1.41 | 0.81 | 0.94 | 0.93 |
| 002088_hsa-miR-636_A | 1.78 | 0.81 | 0.88 | 0.93 |
| 002234_hsa-miR-140-3p_A | 1.33 | 0.83 | 1.06 | 0.95 |
| 002230_hsa-miR-330-5p_A | 1.33 | 0.93 | 1.07 | 0.95 |
| 002282_hsa-let-7g_A | 1.15 | 0.81 | 1.02 | 0.95 |
| 000416_hsa-miR-30a-3p_B | 1.01 | 0.96 | 0.99 | 0.95 |
| 002251_hsa-miR-200b_A | 1.24 | 0.81 | 0.99 | 0.96 |
| 000468_hsa-miR-146a_A | 1.01 | 0.97 | 1.02 | 0.96 |
| 002261_hsa-miR-135b_A | 1.40 | 0.81 | 0.98 | 0.96 |
| 002125_hsa-miR-374a#_B | 2.17 | 0.81 | 0.95 | 0.96 |
| 000433_hsa-miR-95_A | 1.14 | 0.93 | 0.97 | 0.96 |
| 000451_hsa-miR-126#_B | 1.21 | 0.81 | 1.01 | 0.97 |
| 002642_HSA-MIR-151-5P_B | 1.12 | 0.81 | 1.00 | 0.97 |
| 002303_hsa-miR-450a_A | 1.11 | 0.96 | 0.97 | 0.97 |
| 000457_hsa-miR-132_A | 1.08 | 0.81 | 1.00 | 0.98 |
| 002267_hsa-miR-545_A | 1.67 | 0.84 | 1.03 | 0.98 |
| 002228_hsa-miR-126_A | 1.15 | 0.81 | 1.00 | 0.99 |
| 002178_hsa-miR-935_B | 0.82 | 0.81 | 1.00 | 0.99 |
| 000478_hsa-miR-154#_B | 0.68 | 0.81 | 1.00 | 1.00 |

Relative Quantification (RQ)
