# Supplementary material for: Single-walled and multi-walled carbon nanotubes induce sequence-specific epigenetic alterations in 16 HBE cells
Source: Oncotarget. 2018 Apr 17;9(29):20351–65. doi: 10.18632/oncotarget.24866 (PMC5945544; doi:10.18632/oncotarget.24866)
Supplement: Supplementary file 3 [file oncotarget-09-20351-s003.docx]

**Supplementary Table 2**: Results of pyrosequencing assays

|  | | **Control** | **MWCNT (µg/ml)** | | | **SWCNT (µg/ml)** | | | **Decitabine** |
| --- | --- | --- | --- | --- | --- | --- | --- | --- | --- |
|  |  |  | **2.5** | **5** | **25** | **2.5** | **5** | **25** |  |
| **LINE 1** | CpG 1 | 45.23 ± 0.84 | 45.59 ± 0.31 | 45.79 ± 1.46 | 45.21 ± 0.53 | 45.79 ± 0.92 | 45.5 ± 1.16 | 45.42 ± 1.18 | 35.87 ± 0.88 |
|  | CpG 2 | 56.84 ± 0.76 | 57.07 ± 0.11 | 57.45 ± 0.54 | 57.86 ± 1.34 | 57.32 ± 1.01 | 56.94 ± 1.68 | 55.44 ± 0.96 | 42.89 ± 1.17 |
|  | CpG 3 | 57.39 ± 0.24 | 57.76 ± 0.59 | 57.64 ± 0.87 | 57.88 ± 1.04 | 57.97 ± 0.50 | 57.18 ± 1.22 | **55.71 ± 0.87*** | 43.47 ± 0.84 |
|  | Average | 53.15 ± 0.17 | 53.47 ± 0.31 | 53.63 ± 0.51 | 53.65 ± 0.89 | **53.69 ± 0.19*** | 53.21 ± 0.77 | 52.19 ± 0.98 | 40.74 ± 0.54 |
| **SKI** | CpG 1 | 1 ± 0 | 1 ± 0 | 1 ± 0 | 1.33 ± 0.57 | 1.33 ± 0.57 | 1 ± 0 | 1.66 ± 1.15 | 1.33 ± 0.57 |
|  | CpG 2 | 1.66 ± 0.57 | 1 ± 0 | 1.33 ± 0.57 | 1 ± 0 | 1.66 ± 0.57 | 1.66 ± 0.57 | 2.33 ± 2.30 | 1.3 ± 0.57 |
|  | CpG 3 | 2.66 ± 0.57 | 2 ± 1 | 2 ± 0 | 2.33 ± 0.57 | 3 ± 1.73 | 3 ± 1 | 2.66 ± 2.08 | 2.33 ± 0.57 |
|  | CpG 4 | 22 ± 1.73 | 21.33 ± 1.52 | 19.33 ± 1.52 | 20 ± 1 | 20.33 ± 1.15 | 20 ± 1 | 21 ± 2 | 15.66 ± 0.57 |
|  | CpG 5 | 2.66 ± 0.57 | 2.66 ± 0.57 | 2.66 ± 0.57 | 2.33 ± 0.57 | 3 ± 0 | 3 ± 0 | 3 ± 1 | 2.33 ± 0.57 |
|  | CpG 6 | 1.66 ± 0.57 | 1 ± 0 | 1.66 ± 0.57 | 1.33 ± 0.57 | 2 ± 0 | 2 ± 0 | 2 ± 1 | 1.66 ± 0.57 |
|  | Average | 5.27 ± 0.53 | 4.83 ± 0.44 | 4.66 ± 0.28 | 4.72 ± 0.38 | 5.22 ± 0.63 | 5.11 ± 0.19 | 5.44 ± 1.51 | 4.11 ± 0.50 |
| **DNMT 1** | CpG 1 | 0.38 ± 0.12 | 0.45 ± 0.26 | 0.49 ± 0.17 | 0.47 ± 0.25 | 0.77 ± 0.80 | 0.45 ± 0.12 | 0.30 ± 0.05 | 0.73 ± 0.53 |
|  | CpG 2 | 0.46 ± 0.08 | 0.69 ± 0.30 | 0.83 ± 0.39 | 0.53 ± 0.16 | 0.45 ± 0.02 | 0.46 ± 0.10 | **0.81 ± 0.14*** | 0.66 ± 0.14 |
|  | CpG 3 | 0.62 ± 0.22 | 0.45 ± 0.19 | 0.33 ± 0.30 | 0.66 ± 0.30 | 0.54 ± 0.20 | 0.31 ± 0.31 | 0.49 ± 0.20 | 0.79± 0.38 |
|  | CpG 4 | 2.63 ± 0.21 | 3.33 ± 1.17 | 3.11 ± 0.78 | 3.22 ± 0.43 | 2.93 ± 0.88 | 2.64 ± 0.66 | 3.18 ± 0.51 | 2.84 ± 0.53 |
|  | CpG 5 | 0.28 ± 0.14 | 0.22 ± 0.017 | 0.33 ± 0.36 | 0.30 ± 0.04 | 0.35 ± 0.21 | 0.37 ± 0.21 | 0.39 ± 0.16 | 0.44 ± 0.35 |
|  | Average | 0.87 ± 0.06 | 1.028 ± 0.31 | 1.02 ± 0.23 | 1.03 ± 0.15 | 1.01 ± 0.32 | 0.84 ± 0.21 | 1.03 ± 0.09 | 1.09 ± 0.3 |
| **HDAC 4** | CpG 1 | 0.28 ± 0.05 | 0.41 ± 0.11 | 0.42 ± 0.08 | **0.40 ± 0.04*** | 0.406 ± 0.26 | 0.31 ± 0.08 | 0.27 ± 0.14 | 0.37 ± 0.08 |
|  | CpG 2 | 0.32 ± 0.05 | 0.31 ± 0.06 | 0.30 ± 0.08 | 0.23 ± 0.04 | 0.31 ± 0.09 | 0.34 ± 0.08 | 0.30 ± 0.17 | 0.31 ± 0.12 |
|  | CpG 3 | 0.86 ± 0.24 | 0.87 ± 0.56 | 0.73 ± 0.31 | 0.82 ± 0.13 | 0.79 ± 0.34 | 0.61 ± 0.21 | 0.61 ± 0.10 | 0.74 ± 0.12 |
|  | CpG 4 | 1.45 ± 0.14 | 1.46 ± 0.20 | 1.39 ± 0.43 | 1.46 ± 0.30 | 1.45 ± 0.37 | 1.51 ± 0.51 | 1.28 ± 0.09 | 1.70 ± 0.48 |
|  | CpG 5 | 0.69 ± 0.047 | 0.78 ± 0.16 | 0.63 ± 0.12 | 0.72 ± 0.09 | 1.01 ± 0.23 | 0.74 ± 0.21 | 0.60 ± 0.04 | 0.80 ± 0.02 |
|  | CpG 6 | 0.33 ± 0.30 | 0.39 ± 0.27 | 0.21 ± 0.01 | 0.22 ± 0.15 | 0.25 ± 0.24 | 0.33 ± 0.26 | 0.25 ± 0.07 | 0.31 ± 0.15 |
|  | CpG 7 | 0.25 ± 0.23 | 0.26 ± 0.09 | 0.29 ± 0.04 | 0.24 ± 0.05 | 0.14 ± 0.13 | 0.24 ± 0.05 | 0.25 ± 0.04 | 0.26 ± 0.04 |
|  | Average | 0.6 ± 0.04 | 0.64 ± 0.16 | 0.57 ± 0.09 | 0.58 ± 0.05 | 0.62 ± 0.15 | 0.58 ± 0.16 | 0.51± 0.05 | 0.64 ± 0.11 |
| **NPAT/ATM** | CpG 1 | 0.59 ± 0.08 | 0.54 ± 0.26 | 0.59 ± 0.26 | 0.60 ± 0.16 | 0.56 ± 0.58 | 0.51 ± 0.19 | **0.31 ± 0.10*** | 0.53 ± 0.31 |
|  | CpG 2 | 0.53 ± 0.26 | 0.51 ± 0.32 | 0.68 ± 0.39 | 0.45 ± 0.09 | 0.46 ± 0.41 | 0.24 ± 0.04 | 0.42± 0.22 | 0.56 ± 0.46 |
|  | CpG 3 | 0.48 ± 0.17 | 0.86 ± 0.55 | 0.66 ± 0.32 | 0.69 ± 0.34 | 0.81 ± 0.35 | 0.48 ± 0.27 | 0.42 ± 0.20 | 0.46 ± 0.27 |
|  | CpG 4 | 0.28 ± 0.08 | 0.95 ± 0.63 | 0.97 ± 0.59 | 0.50 ± 0.23 | **1.29 ± 0.21*** | 0.31 ± 0.026 | 0.43 ± 0.29 | 0.65 ± 0.23 |
|  | CpG 6 | 1.44 ± 0.30 | 2.05 ± 0.88 | 2.44 ± 0.15 | 1.75 ± 0.16 | **2.52 ± 0.50*** | 1.92 ± 0.24 | 1.69 ± 0.05 | 2.03 ± 0.49 |
|  | Average | 0.66 ± 0.13 | 0.98 ± 0.48 | 1.07 ± 0.27 | 0.80 ± 0.08 | **1.13 ± 0.08*** | 0.69 ± 0.07 | 0.65 ± 0.09 | 0.84 ± 0.34 |
| **BCL2L11** | CpG 1 | 0.33 ± 0.57 | 0.66 ± 0.57 | 0.33 ± 0.57 | 1 ± 0 | 0.66 ± 0.57 | 0 ± 0 | 0.33 ± 0.57 | 0.33 ± 0.57 |
|  | CpG 2 | 0.33 ± 0.57 | 0.33 ± 0.57 | 0.66 ± 0.57 | 0 ± 0 | 0.33 ± 0.57 | 0 ± 0 | 0 ± 0 | 0.66 ± 0.57 |
|  | CpG 3 | 0.66 ± 0.57 | 1 ± 0 | 1 ± 0 | 1 ± 0 | 1.33 ± 0.57 | 1 ± 0 | 1 ± 0 | 1 ± 0 |
|  | CpG 4 | 1 ± 1 | 1 ± 1 | 1 ± 0 | 1.33 ± 0.57 | 2 ± 0 | 1.33 ± 0.57 | 1 ± 0 | 1 ± 0 |
|  | CpG 5 | 1 ± 0 | 1.66 ± 0.57 | 1.33 ± 0.57 | 1 ± 0 | 1.66 ± 1.15 | 1.66 ± 0.57 | 1.66 ± 0.57 | 1 ± 0 |
|  | CpG 7 | 1 ± 0 | 1 ± 0 | 1 ± 0 | 1 ± 0 | 1.33 ± 0.57 | 1 ± 0 | 1 ± 0 | 1 ± 0 |
|  | Average | 0.72 ± 0.34 | 0.94 ± 0.19 | 0.88 ± 0.09 | 0.88± 0.09 | 1.22 ± 0.41 | 0.83 ± 0 | 0.83 ± 0.16 | 0.8 ± 0.16 |
| **MAP3K10** | CpG 1 | 6.16 ± 0.58 | **5.03 ± 0.27*** | **4.48 ± 0.14*** | 5.01 ± 0.58 | 5.77 ± 1.56 | 5.95 ± 0.30 | 7.44± 1.30 | 7.19 ± 0.25 |
|  | CpG 2 | 0.89 ± 0.15 | 0.75 ± 0.14 | 1.69 ± 1.10 | 0.86 ± 0.06 | 0.72 ± 0.08 | 0.74 ± 0.41 | 0.86 ± 0.25 | 1.02 ± 0.30 |
|  | CpG 3 | 8.24 ± 0.68 | 7.43 ± 0.74 | **6.64 ± 0.41*** | **6.33 ± 0.56*** | 7.70 ± 1.33 | 8.84 ± 0.42 | 9.65 ± 1.33 | 9.29 ± 0.32 |
|  | CpG 4 | 10.06 ± 0.93 | 8.94 ± 0.54 | 8.39 ± 0.53 | **7.54 ± 0.69*** | 9.12 ± 1.55 | 11.08 ± 0.44 | 11.74 ± 2.08 | 11.05 ± 0.77 |
|  | CpG 5 | 9.97 ± 0.70 | 9.35 ± 0.52 | 8.83 ± 0.24 | **7.80 ± 0.65*** | 9.56 ± 2.12 | 11.03 ± 0.58 | 12.03 ± 1.60 | 11.36 ± 0.49 |
|  | CpG 6 | 1.33 ± 0.17 | 2.03 ± 0.63 | **1.42 ± 0.05*** | 1.54 ± 0.27 | 1.46± 0.08 | 2.03 ± 0.50 | 1.72 ± 0.40 | 2.02 ± 0.50 |
|  | Average | 6.11 ± 0.40 | 5.59 ± 0.27 | **5.24 ± 0.38*** | **4.84 ± 0.42*** | 5.725 ± 1.08 | 6.61± 0.40 | 7.24 ± 0.99 | 6.99 ± 0.20 |
| **PIK3R2** | CpG 1 | 0.36 ± 0.04 | 0.55± 0.46 | 0.56 ± 0.22 | 0.41 ± 0.11 | 0.56 ± 0.24 | 0.57 ± 0.21 | 0.45 ± 0.21 | 1.13 ± 1.34 |
|  | CpG 2 | 0.46 ± 0.08 | 0.66 ± 0.43 | 0.72 ± 0.24 | 0.71 ± 0.17 | 0.77 ± 0.34 | 0.57 ± 0.16 | 0.42 ± 0.02 | 1.09 ± 1.19 |
|  | CpG 3 | 0.41 ± 0.18 | 0.39 ± 0.15 | 0.29 ± 0.11 | 0.28 ± 0.10 | 0.16 ± 0.13 | 0.30 ± 0.08 | 0.20 ± 0.02 | 0.70 ± 0.61 |
|  | CpG 4 | 0.71 ± 0.14 | 1.11 ± 0.45 | 0.89 ± 0.32 | 0.97 ± 0.18 | 1.15 ± 0.22 | 0.82 ± 0.19 | 0.80 ± 0.18 | 1.18 ± 0.66 |
|  | CpG 5 | 0.64 ± 0.15 | 1.26 ± 0.60 | 0.96 ± 0.15 | 0.85 ± 0.25 | 1.44 ± 0.75 | 0.70 ± 0.11 | 0.56 ± 0.06 | 1.68 ± 1.23 |
|  | Average | 0.51 ± 0.02 | 0.79 ± 0.38 | 0.68 ± 0.06 | **0.64 ± 0.02*** | 0.81 ± 0.24 | **0.59 ± 0.04*** | **0.48 ± 0.009*** | 1.16 ± 1.00 |
| **MYO1C** | CpG 1 | 60.16 ± 3.57 | 57.47 ± 7.95 | 60.44 ± 0.95 | 59.51 ± 2.24 | 54.79 ± 9.31 | **68.54 ± 3.45*** | 60.46 ± 3.96 | 49.15 ± 4.22 |
|  | CpG 2 | 73.5 ± 1.44 | 71.02 ± 6.06 | 75.70 ± 3.90 | 73.67 ± 4.80 | 71.02 ± 4.37 | 81.91 ± 5.26 | 71.52 ± 4.01 | 60.86 ± 5.02 |
|  | CpG 3 | 63.45± 0.55 | 60.11 ± 7.39 | 62.58 ± 4.24 | 62.04± 5.04 | 60.62 ± 8.26 | 70.59 ± 5.13 | 62.95 ± 3.07 | 51.54 ± 6.01 |
|  | CpG 5 | 68.47 ± 2.13 | 64.22 ± 8.20 | 69.5 ± 3.69 | 65.15 ± 3.49 | 66.61 ± 10.62 | 77.44 ± 4.492 | 66.34 ± 2.03 | 57.13 ± 6.49 |
|  | CpG 6 | 58.4 ± 2.84 | 54.28 ± 5.91 | 57.95 ± 2.69 | 56.71 ± 4.65 | 55.97 ± 6.75 | 62.96 ± 2.94 | 56.17 ± 2.76 | 47.17 ± 4.74 |
|  | Average | 64.81 ± 1.24 | 61.42 ± 6.96 | 65.23 ± 3.05 | 63.41 ± 3.97 | 61.80 ± 7.78 | **72.29 ± 4.15*** | 63.49 ± 2.99 | 53.17 ± 5.18 |
| **TCF3** | CpG 1 | 1.13 ± 1.42 | 0.25 ± 0.01 | 0.42 ± 0.22 | 0.34 ± 0.41 | 0.65± 0.40 | 0.31 ± 0.16 | 0.47 ± 0.35 | 0.30 ± 0.05 |
|  | CpG 2 | 0.85 ± 0.28 | 0.61 ± 0.28 | 0.96± 0.60 | 0.51 ± 0.20 | 0.50 ± 0.09 | 0.78 ± 0.44 | 0.48 ± 0.13 | 0.53 ± 0.09 |
|  | CpG 3 | 0.37 ± 0.11 | 0.4 ± 0.23 | 0.30 ± 0.09 | 0.24 ± 0.26 | 0.7 ± 0.47 | 0.48 ± 0.35 | 0.20 ± 0.18 | 0.37 ± 0.08 |
|  | CpG 4 | 0.80 ± 0.40 | 0.96 ± 0.14 | 0.91 ± 0.51 | 1.04 ± 0.40 | 1.42 ± 1.17 | 0.77 ± 0.56 | 0.53 ± 0.30 | 0.78 ± 0.18 |
|  | Average | 0.79 ± 0.52 | 0.55 ± 0.01 | 0.65 ± 0.25 | 0.53 ± 0.10 | 0.81 ± 0.43 | 0.59 ± 0.33 | 0.42 ± 0.13 | 0.49 ± 0.06 |
| **FGFR 1** | CpG 1 | 2.38 ± 0.50 | 1.64 ± 1.32 | 2.07 ± 0.94 | 1.86 ± 0.39 | 1.72 ± 0.12 | 2.38 ± 1.25 | 2.90 ± 0.33 | 1.80 ± 0.55 |
|  | CpG 2 | 2.62 ± 0.08 | 2.31 ± 0.71 | 2.40 ± 0.37 | 2.20 ± 0.68 | 2.35 ± 1.16 | 3.01± 0.44 | 2.63 ± 0.44 | 2.27 ± 0.34 |
|  | CpG 3 | 13.22 ± 1.02 | 13.91 ± 0.91 | 14.26± 1.93 | 14.19 ± 2.66 | 14.43 ± 2.63 | 11.85 ± 0.66 | 14.56 ± 0.92 | 10.57 ± 1.21 |
|  | Average | 6.07 ± 0.15 | 5.95 ± 0.47 | 6.24 ± 1.01 | 6.085 ± 0.76 | 6.16 ± 0.79 | 5.74 ± 0.19 | 6.70 ± 0.36 | 4.88 ± 0.46 |
| **AGRN** | CpG 1 | 93.33 ± 0.57 | 93 ± 1.73 | 93 ± 1 | 93.66 ± 0.57 | 93.33 ± 0.57 | 93.33 ± 0.57 | 94.33 ± 0.57 | 74.33 ± 0.57 |
|  | CpG 2 | 95.66 ± 0.57 | 95.66 ± 0.57 | 95.33 ± 0.57 | 96 ± 0 | 95 ± 0 | 95.33 ± 0.57 | 96.66 ± 0.57 | 76.66 ± 1.15 |
|  | CpG 3 | 96.33 ± 0.57 | 96 ± 1 | 96.33 ± 0.57 | 96 ± 1 | 97 ± 0 | 96.66± 0.57 | 96.33 ± 1.15 | 78.66 ± 0.57 |
|  | CpG 4 | 98.33 ± 1.15 | 99 ± 1 | 96.66 ± 0.57 | 97.66± 1.52 | 97.33 ± 0.57 | 98 ± 1 | 97.66 ± 0.57 | 78 ± 1 |
|  | CpG 5 | 95 ± 1 | 95.33 ± 1.52 | 95.33 ± 0.57 | 95.66 ± 0.57 | 95 ± 1 | 95.33 ± 0.57 | 94.33 ± 0.57 | 77.66 ± 0.57 |
|  | Average | 80.65 ± 0.10 | 80.63 ± 0.50 | 80.22 ± 0.24 | 80.62 ± 0.26 | 80.48 ± 0.35 | 80.62 ± 0.53 | 80.79 ± 0.42 | 64.90 ± 0.06 |

* P < 0.05, Values in red represent statistically significant values, results of positive control (Decitabine) have not been included in the statistical representation
